# Identification of the lamin A/C phosphoepitope recognized by the antibody P-STM in mitotic HeLa S3 cells

**DOI:** 10.1186/1471-2091-14-18

**Published:** 2013-07-19

**Authors:** Jeng-Ting Chen, Chia-Wen Ho, Lang-Ming Chi, Kun-Yi Chien, Ya-Ju Hsieh, Shih-Jie Lin, Jau-Song Yu

**Affiliations:** 1 Graduate Institute of Biomedical Sciences, College of Medicine, Chang Gung University, Tao-Yuan, Taiwan, Republic of China; 2 Department of Cell and Molecular Biology, College of Medicine, Chang Gung University, Tao-Yuan, Taiwan, Republic of China; 3 Department of Medical Research and Development, Chang Gung Memorial Hospital, Tao-Yuan, Taiwan, Republic of China; 4 Molecular Medicine Research Center, Chang Gung University, Tao-Yuan, Taiwan, Republic of China

**Keywords:** P-STM antibody, Phosphoepitope, Lamin A/C, Mitosis, SILAC

## Abstract

**Background:**

Lamins A and C, two major structural components of the nuclear lamina that determine nuclear shape and size, are phosphoproteins. Phosphorylation of lamin A/C is cell cycle-dependent and is involved in regulating the assembly–disassembly of lamin filaments during mitosis. We previously reported that P-STM, a phosphoepitope-specific antibody raised against the autophosphorylation site of p21-activated kinase 2, recognizes a number of phosphoproteins, including lamins A and C, in mitotic HeLa cells.

**Results:**

Here, using recombinant proteins and synthetic phosphopeptides containing potential lamin A/C phosphorylation sites in conjunction with *in vitro* phosphorylation assays, we determined the lamin A/C phosphoepitope(s) recognized by P-STM. We found that phosphorylation of Thr-19 is required for generating the P-STM phosphoepitope in lamin A/C and showed that it could be created *in vitro* by p34^cdc2^/cyclin B kinase (CDK1)-catalyzed phosphorylation of lamin A/C immunoprecipitated from unsynchronized HeLa S3 cells. To further explore changes in lamin A/C phosphorylation in living cells, we precisely quantified the phosphorylation levels of Thr-19 and other sites in lamin A/C isolated from HeLa S3 cells at interphase and mitosis using the SILAC method and liquid chromatography-tandem mass spectrometry. The results showed that the levels of phosphorylated Thr-19, Ser-22 and Ser-392 in both lamins A and C, and Ser-636 in lamin A only, increased ~2- to 6-fold in mitotic HeLa S3 cells.

**Conclusions:**

Collectively, our results demonstrate that P-STM is a useful tool for detecting Thr-19-phosphorylated lamin A/C in cells and reveal quantitative changes in the phosphorylation status of major lamin A/C phosphorylation sites during mitosis.

## Background

The nuclear envelope consists of inner and outer nuclear membranes, nuclear pore complexes, and the underlying nuclear lamina. The nuclear lamina connects with both integral membrane proteins of the inner nuclear membrane and chromatin [1]. Nuclear lamins are intermediate filament proteins that are the major components of the nuclear lamina. Lamins are type V intermediate filament proteins consisting of a central coiled-coil region and globular N- and C-terminal domains. They are classified into two subgroups: A-type and B-type. A-type lamins, including lamin A (74 kDa) and lamin C (65 kDa), are alternatively spliced products of a single gene locus, with lamin A having an extra 98 amino acid residues at its C-terminus that are not present in lamin C [1,2]. During the mitotic phase of every cell cycle in eukaryotes, the structure of the nuclear envelope undergoes a dramatic assembly and disassembly process. The nuclear lamina depolymerizes as a result of mitosis-specific phosphorylation of the nuclear lamins at specific sites [3,4]. Several kinases, including p34^cdc2^/cyclin B (CDK1), CDK5, protein kinase C (PKC) and Akt/protein kinase B (PKB), are reported to phosphorylate lamin A/C on different sites during mitosis or under certain physiological/pathological conditions [3-9]. Multiple cell cycle-dependent lamin C phosphorylation sites have been determined [5]. Mutation of two lamin A phosphorylation sites, Ser-22 (in QASSTPLS^22^PTRIT) and Ser-392 (in RLRLSPS^392^PTSQR), has been reported to prevent nuclear lamina disassembly in mitotic cells [3].

Phosphoepitope-specific antibodies that selectively recognize phosphorylated, but not non-phosphorylated, forms of a variety of protein kinases or their substrates are valuable tools for measuring kinase activity and substrate phosphorylation during numerous cellular events [10-13]. Many of these antibodies are generated in animals using a specific phosphopeptide as the antigen. Because the phosphate group is a common structure present in every phosphopeptide antigen, an anti-phosphopeptide antibody raised against a specific phosphopeptide may display a broader immunoreactivity, interacting with structurally similar phosphorylated motifs [14-17].

PAK2, a member of the p21-activated kinase (PAK) family, participates in regulating diverse cell functions, including cell morphogenesis, motility, survival, apoptosis, mitosis, and angiogenesis [18,19]. Previously, we identified Thr-402 as the autophosphorylation regulatory site of the catalytic fragment of PAK2 [20]. We subsequently generated a phosphoepitope-specific antibody, P-STM, using the synthetic phospho-STM-11-C peptide, S^398^KRSTp^402^MVGTPYC^408^, which encompasses the sequence of the regulatory autophosphorylation site of PAK2, as an antigen. This antibody recognizes the autophosphorylated/activated, but not the unphosphorylated/inactive, form of PAK2 [20]. Interestingly, we found that P-STM also reacted with a number of mitotic phosphoproteins in human A431 and HeLa cells [21] that are different from those detected by MPM-2, a monoclonal antibody raised against total lysates of mitotic HeLa cells that recognizes more than 40 mitotic phosphoproteins [22,23]. Using two-dimensional gel electrophoresis and matrix-assisted laser desorption/ionization-time of flight (MALDI-TOF) mass spectrometry, we further identified lamins A and C as two of the mitotic cell-specific phosphoproteins recognized by P-STM [21]. However, the exact lamin A/C phosphoepitope(s) recognized by P-STM, the kinase responsible for generating the phosphoepitope(s), as well as quantitative changes in the phosphoepitope(s) between interphase and mitosis, remained elusive.

The aims of this study were to identify the lamin A/C phosphoepitope(s) recognized by P-STM in mitosis and quantify differential phosphorylation of lamin A/C phosphorylation sites at interphase and mitosis using liquid chromatography-tandem mass spectrometry (LC-MS/MS) coupled with the SILAC (stable isotope labeling by amino acids in cell culture) approach. We found that CDK1-catalyzed phosphorylation of lamin A/C at Thr-19 created a P-STM-recognizable phosphoepitope. SILAC-based quantitative mass spectrometric analyses of lamin A/C isolated from HeLa S3 cells at interphase and mitosis further revealed 2.3- to 5.6-fold increases in the phosphorylation levels of Thr-19, Ser-22 and Ser-392 in lamins A and C, and Ser-636 in lamin A only, in mitotic compared with interphase HeLa S3 cells. Among the four sites identified and quantified, Thr-19 and Ser-22 showed the most dramatic cell cycle-dependent changes in phosphorylation.

## Results

### Phosphorylation of interphase lamin A/C by CDK1 Creates a P-STM phosphoepitope

**Figure 1:**
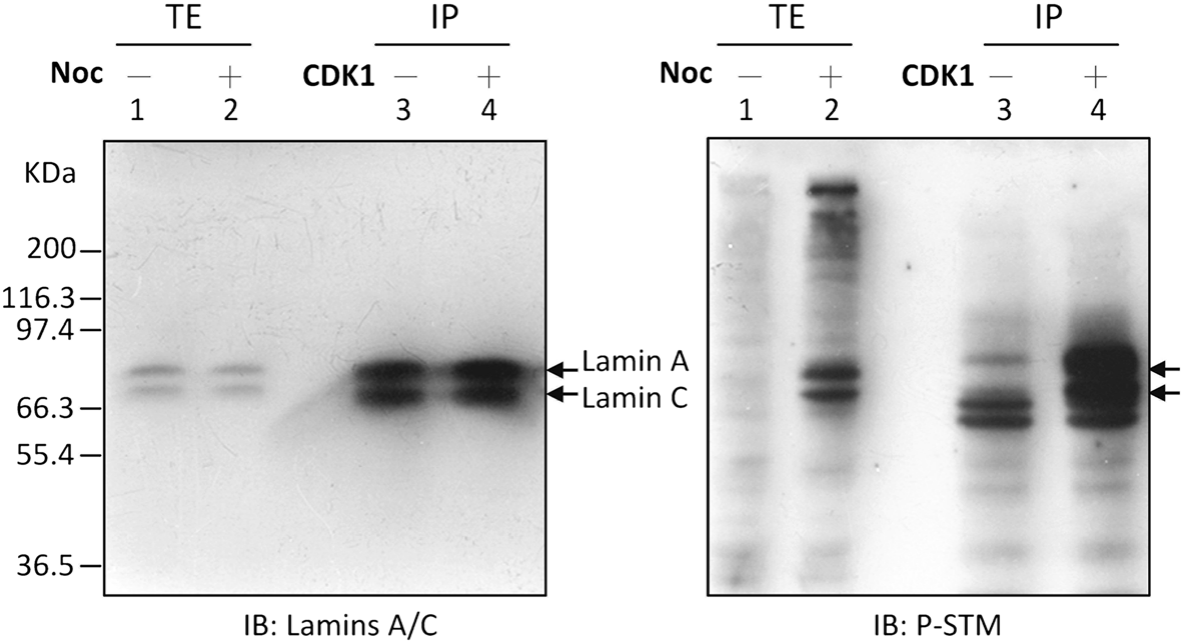
**CDK1-catalyzed phosphorylation of interphase lamins A and C enhances their P-STM immunoreactivity.** Total extracts (TE; 60 μg) from HeLa S3 cells treated for 14 h with DMSO (lane 1) or Noc (1 μg/mL; lane 2) were resolved by SDS-PAGE on 10% gels and immunoblotted (IB) with P-STM antibody or anti-lamin A/C antibody. Lamins A and C were immunoprecipitated (IP) from total extracts of unsynchronized cells (2 mg) and then *in vitro*-phosphorylated by incubating with CDK1 (lane 4) for 30 min; immunoprecipitates incubated without CDK1 (lane 3) served as controls. IP products were resolved by SDS-PAGE on 10% gels and analyzed by immunoblotting as described.

We have previously shown that lamins A and C are two of the mitotic phosphoproteins recognized by the P-STM antibody [21]. Because the CDK1 complex is known to phosphorylate lamin A/C at multiple sites during mitosis [3-6], we tested whether interphase lamin A/C can be phosphorylated *in vitro* by CDK1 to create a P-STM phosphoepitope. Lamins A and C were immunoprecipitated from total extracts of unsynchronized HeLa S3 cells and *in vitro*-phosphorylated by CDK1. The reaction products, as well as total extracts of HeLa S3 cells, treated without or with nocodazole (Noc), were then immunoblotted with anti-lamin A/C antibody or P-STM. Both lamins A and C were efficiently immunoprecipitated and detected with anti-lamin A/C antibody (Figure 1, left panel). After *in vitro* CDK1-catalyzed phosphorylation of immunoprecipitated lamin A/C, two major additional P-STM-immunoreactive signals corresponding to phosphorylated lamins A and C emerged (Figure 1, right panel; compare lanes 3 and 4), indicating that CDK1-mediated phosphorylation of interphase lamins A and C generates P-STM-recognizable phosphoepitope(s) *in vitro*.

### *In vitro* phosphorylation of recombinant GST-Lamin A/C by CDK1 creates a P-STM phosphoepitope

**Figure 2:**
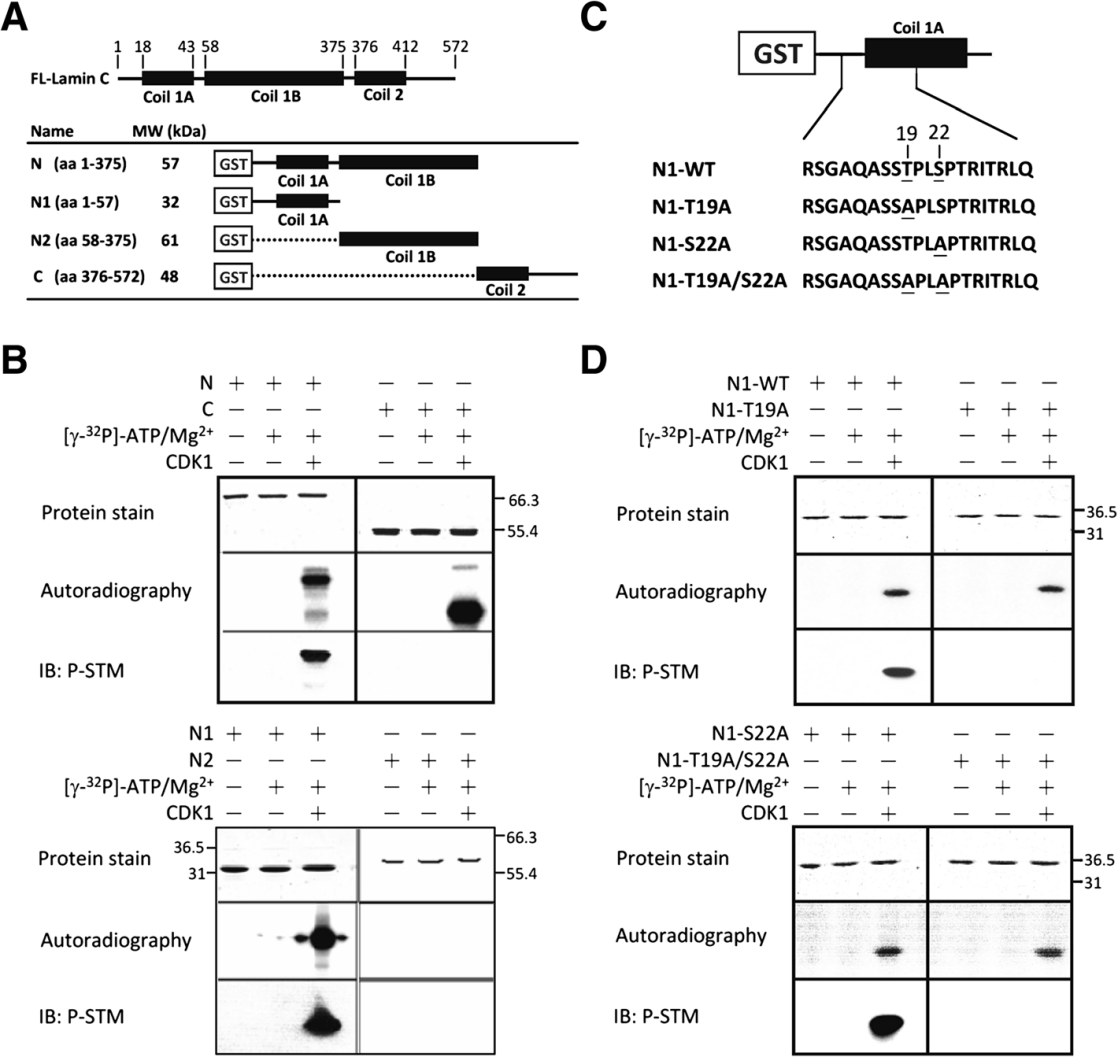
**Identification of CDK1-catalyzed phosphorylation site(s) on lamin A/C as P-STM phosphoepitope(s). (A)** A schematic diagram of full-length lamin C (aa 1–572) and different truncated forms (N, N1, N2, and C) of GST-lamin C. **(B)** The four purified GST-lamin C recombinant proteins (1.5 μg) were *in vitro*-phosphorylated by CDK1 in the presence of [γ-^32^P]-ATP. Reaction products were resolved by SDS-PAGE on 10% gels and visualized by Coomassie Blue staining (upper panel), followed by autoradiography (middle panel) or immunoblotting with P-STM antibody (lower panel). **(C)** A schematic diagram of GST-lamin C-N1 mutants. Mutants were generated by site-directed mutagenesis to replace Thr-19, Ser-22, or Thr-19/Ser-22 with Ala. **(D)** The four purified GST-lamin C-N1 fusion proteins (1.5 μg) were phosphorylated by CDK1 and processed as described above.

To identify the CDK1-dependent phosphorylation site(s) on lamin A/C recognized by P-STM, we performed *in vitro* phosphorylation assays using bacterially expressed, recombinant GST-lamin A/C fusion proteins as substrates for CDK1. These fusion proteins cover different domains (N, amino acids [aa] 1–375 covering Coil 1A and 1B domains; N1, aa 1–57 covering Coil 1A domain; N2, aa 68–375 covering Coil 1B domain; and C, aa 376–572 covering Coil 2 domain and the nuclear localization signal) of lamin C (Figure 2A) containing known phosphorylation sites for CDK1 (Thr-19, Ser-22, Ser-390, and Ser-392), PKC (Ser-403 and Ser-404), or Akt/PKB (Ser-404). The reaction products were separated by sodium dodecyl sulfate-polyacrylamide gel electrophoresis (SDS-PAGE) and subjected to protein staining, autoradiography, and immunoblotting with P-STM **(**Figure 2B**)**. Although radiography revealed that the N-terminal region (aa 1–375) and the smaller N1 region (aa 1–57) within it, as well as the C-terminal region (aa 376–572), were strongly phosphorylated by CDK1, a P-STM phosphoepitope was created by CDK1 only in the intact N-terminal and N1 regions. These results indicate that the CDK1-dependent P-STM phosphoepitope is located within the N1 region (aa 1–57) of lamin A/C.

### CDK1-mediated Thr-19 phosphorylation of lamin A/C produces a P-STM phosphoepitope

**Figure 3:**
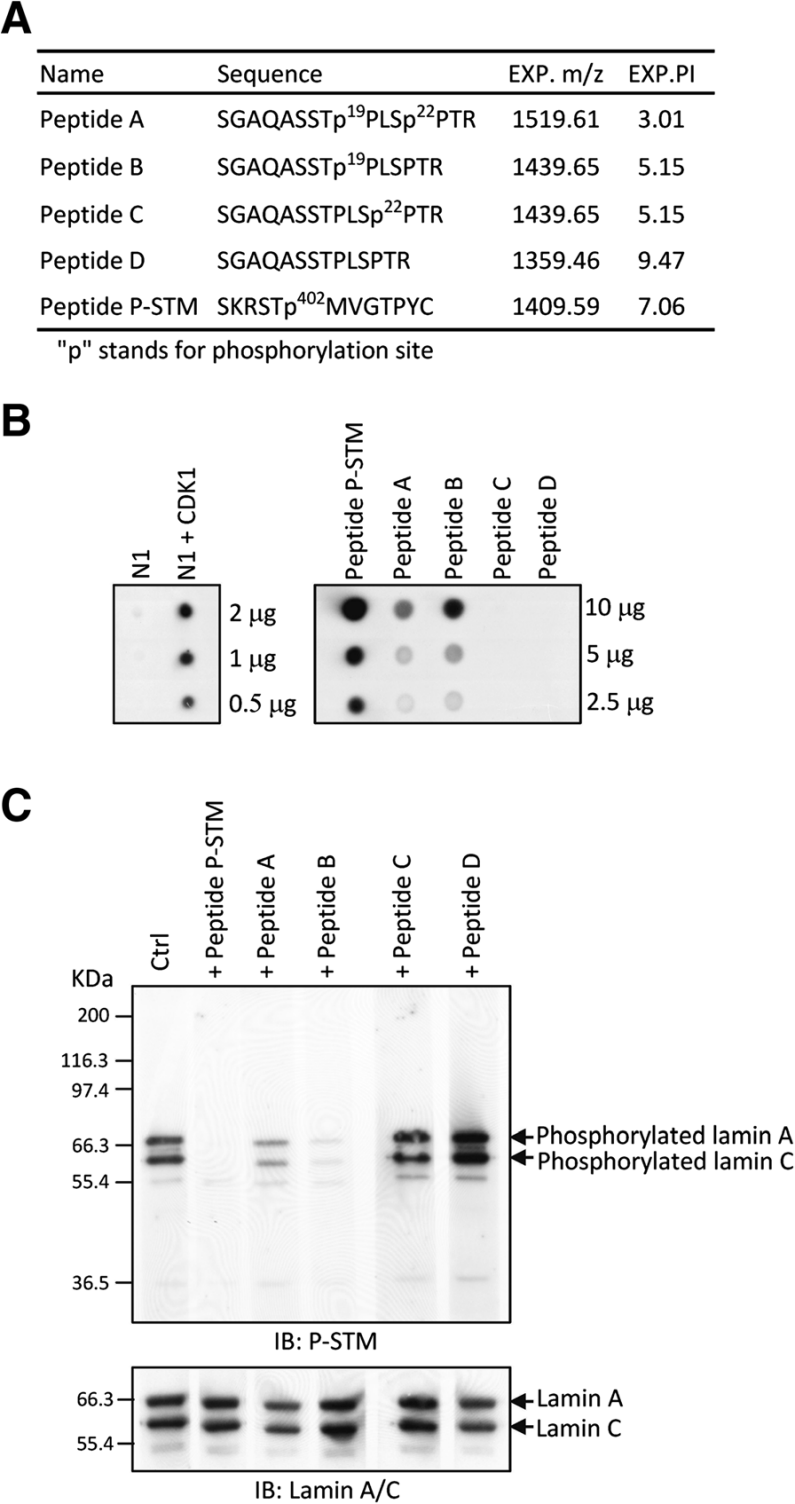
**Mapping the P-STM phosphoepitope on lamin A/C using synthetic phosphopeptides. (A)** Sequences and properties of the synthetic peptides used in this study. **(B)** GST-lamin C-N1 protein (0.5–2 μg), phosphorylated without or with CDK1, and synthetic peptides (10 μg) were subjected to dot-blot analysis with the P-STM antibody. **(C)** Lamins A and C were immunoprecipitated from total extracts (1 mg) of Noc-treated HeLa S3 cells and resolved by SDS-PAGE on 10% gels. *Upper panel:* In competition assays, the IP products were analyzed by immunoblotting with P-STM antibody in the presence of the indicated peptide (50 μg/mL). *Lower panel:* Immunoblot analysis of the same IP products using an anti-lamin A/C antibody (loading control).

In the N1 region (aa 1–57) of lamin A/C, two residues (Thr-19 and Ser-22) are known to be phosphorylated by CDK1 during mitosis [4]. Taken together with the data shown above, this suggests that phosphorylation of Thr-19 and/or Ser-22 by CDK1 may create the P-STM phosphoepitope. To test this hypothesis, we replaced Thr-19 and/or Ser-22 in the N1 region of lamin A/C with Ala by site-directed mutagenesis, and expressed and purified these mutated GST-fusion proteins (N1-T19A, N1-S22A, and N1-T19A/S22A) (Figure 2C). These recombinant proteins were *in vitro-*phosphorylated by CDK1, and the reaction products were analyzed as described above. The results showed that mutation of Thr-19, but not Ser-22, to Ala completely blocked formation of the P-STM phosphoepitope, although all three mutated proteins could still be phosphorylated by CDK1 (Figure 2D). In addition, dot-blot analyses using synthetic phosphopeptides A–D (Figure 3A), which mimic the tryptic digestion products of phosphorylated lamin A/C containing aa 12–25, were further performed to confirm the P-STM phosphoepitope. These dot-blot experiments clearly showed that the P-STM antibody could detect both the CDK1-phosphorylated GST–N1 fusion protein and the authentic P-STM peptide antigen used to generate the P-STM antibody (Figure 3B). Under the assay conditions used, we found that the P-STM antibody recognized peptide B (SGAQASSTp^19^PLSPTR) and, to a lesser extent, peptide A (SGAQASSTp^19^PLSp^22^PTR), but not peptides C (SGAQASSTPLSp^22^PTR) or D (SGAQASST^19^PLS^22^PTR) (Figure 3B). Moreover, the immunoreactivity of the P-STM antibody toward lamins A and C immunoprecipitated from Noc-treated HeLa S3 cells was significantly blocked by inclusion of peptide B and, to a lesser extent, peptide A, but not peptide C or D, in immunoblot competition assays (Figure 3C). Taken together, these results indicate that phosphorylation of Thr-19 in lamin A/C by CDK1 during mitosis creates the P-STM phosphoepitope.

### LC-MS/MS-based identification of phospho-Thr-19 and/or -Ser-22-containing peptides derived from a GST–N1 fusion protein phosphorylated by CDK1 *in vitro*

**Figure 4:**
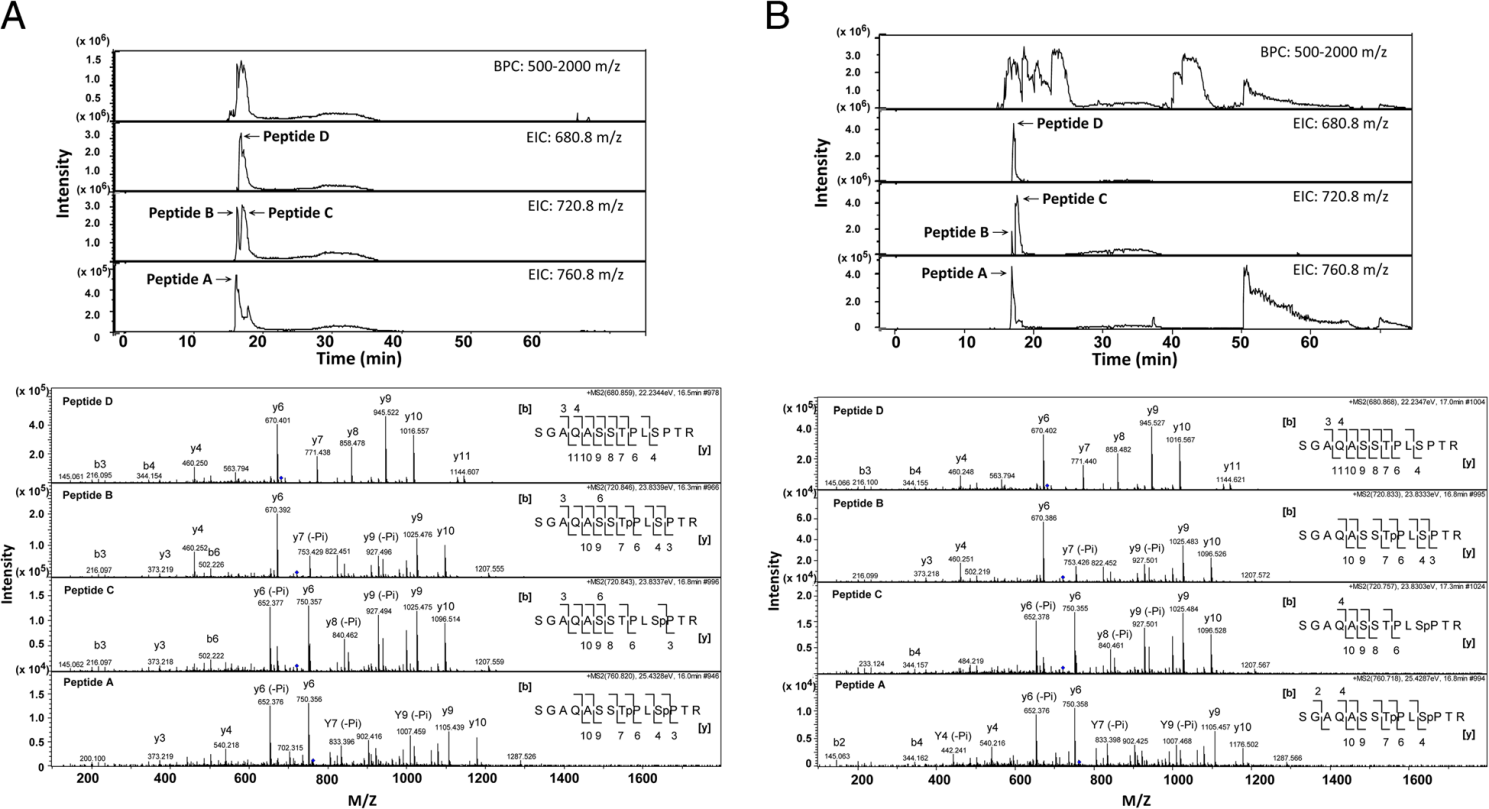
**LC-MS/MS analysis of synthetic phosphopeptides and tryptic peptides derived from GST–N1 fusion protein phosphorylated by CDK1 *in vitro*. (A)** A mixture of the four synthetic peptides (A–D, 6 pmol each) was separated and detected simultaneously by LC-MS/MS using micrOTOFq. *Upper panel:* Base peak chromatogram (BPC) and extracted ion chromatogram (EIC) of a single LC run. *Lower panel:* MS/MS spectra of the four peptides used to assign amino acid sequences. **(B)** The tryptic peptides derived from the GST–N1 fusion protein (0.2 μg, 6.25 pmol) phosphorylated by CDK1 *in vitro* were separated and detected by LC-MS/MS using micrOTOFq. *Upper panel:* BPC and EIC of a single LC run; tryptic peptides corresponding to peptide A-D are highlighted. *Lower panel:* MS/MS spectra of the four tryptic peptides used to assign amino acid sequences.

We have shown that phosphorylation of Thr-19 in lamin A/C by CDK1 can generate a P-STM phosphoepitope and that the P-STM antibody can recognize both mono-phosphorylated peptide B (SGAQASSTp^19^PLSPTR) and dual-phosphorylated peptide A (SGAQASSTp^19^PLSp^22^PTR). However, it is not clear whether the mono-phosphopeptide (peptide B) or dual phosphopeptide (peptide A) form produced by CDK1-catalyzed phosphorylation of lamin A/C is the major contributor to the formation of the P-STM-immunoreactive site. To address this issue, we applied LC-MS/MS to directly detect peptides A–D in tryptic digestion products of GST–N1 fusion protein phosphorylated by CDK1 *in vitro*. First, equal amounts of the four model synthetic peptides A–D (6 pmol each) were mixed and applied to LC-MS/MS to generate standard chromatograms and MS/MS spectra for comparison. As shown in Figure 4A (upper panel), peptides A, B and D coeluted almost together, followed immediately by peptide C; the rank order of signal intensity of these peptides was peptide D > peptide C > peptide B > > peptide A. Subsequent MS/MS analysis of each target peptide based on its known mass and MS/MS spectral assignments unambiguously identified all four peptide standards in one LC-MS/MS run (Figure 4A and Additional file 1: Figure S1). Interestingly, although peptides B (SGAQASSTp^19^PLSPTR) and C (SGAQASSTPLSp^22^PTR) are mono-phosphopeptide isomers with the same mass and isoelectric point (Figure 3A), they could still be separated and unambiguously identified under the LC-MS/MS conditions used here.

We then used these assay conditions to analyze the tryptic peptides derived from the GST–N1 fusion protein (6 pmol) that had been phosphorylated by CDK1 *in vitro*. All four peptide forms were clearly detected by LC-MS/MS analysis (Figure 4B and Additional file 1: Figure S2), and the patterns of MS/MS spectra for the four peptides obtained from this *in vitro*-phosphorylated sample were almost identical to those of synthetic peptide standards (see lower panels of Figure 4 for comparison). Importantly, both mono-phosphopeptide C (SGAQASSTPLSp^22^PTR) and di-phosphopeptide A (SGAQASSTp^19^PLSp^22^PTR) forms were present in larger amounts than the mono-phosphopeptide B (SGAQASSTp^19^PLSPTR) form (Figure 4B, upper panel). Collectively, these results indicate that CDK1 exhibits multiple phosphorylation modes towards the N-terminal region of lamin A/C and suggest that dual phosphorylation of lamin A/C on Thr-19 and Ser-22 makes a major contribution to the formation of the P-STM-immunoreactive site.

### SILAC-based quantification of differences in levels of site-specific lamin A and C phosphorylation between interphase and mitosis

**Figure 5:**
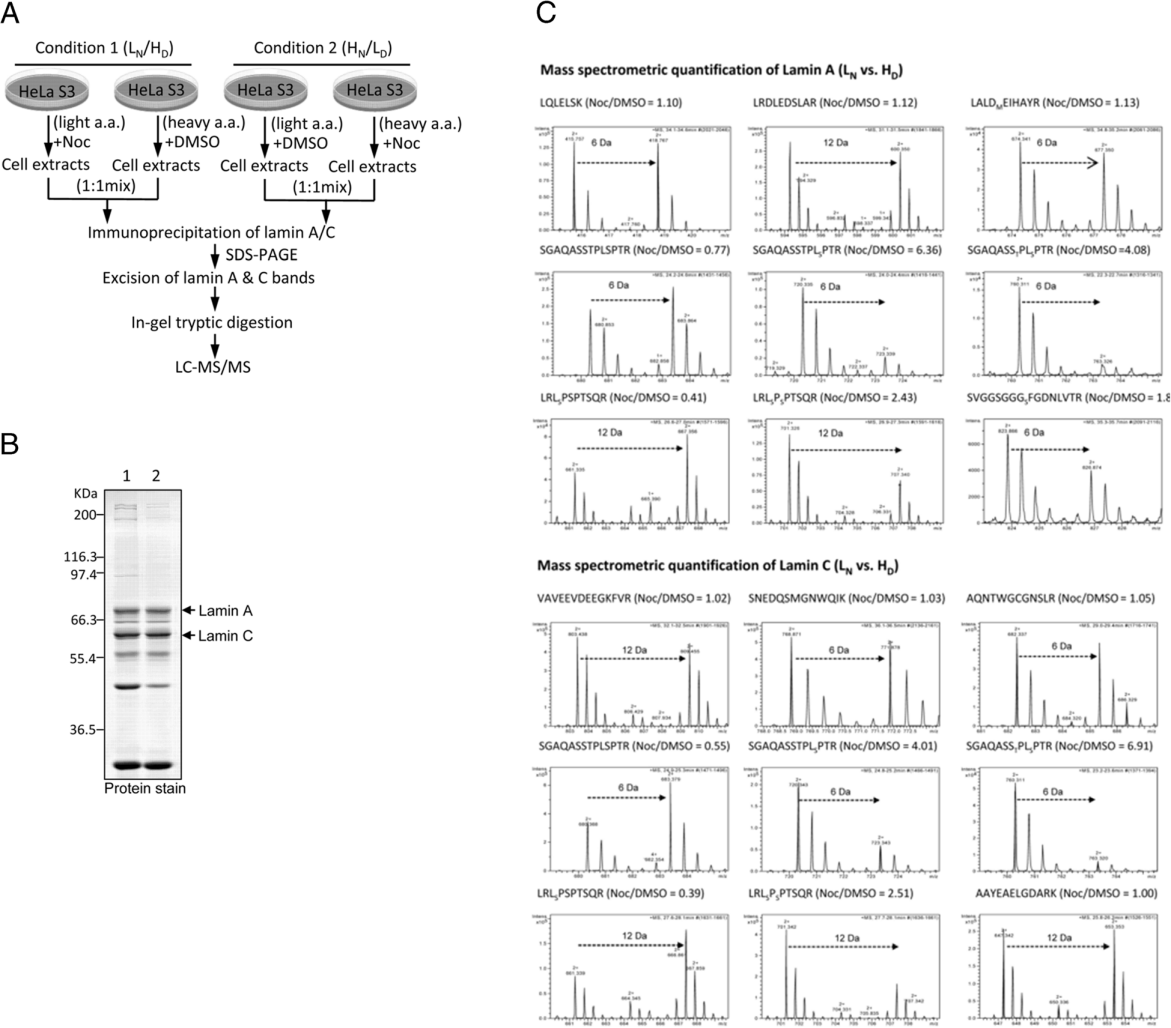
**SILAC-based quantification of tryptic peptides of lamins A and C immunoprecipitated from HeLa S3 cells at interphase and mitosis. (A)** Workflow of the SILAC experiment. L_D_ and L_N_, light isotope-labeled cells treated with DMSO and Noc, respectively; H_D_ and H_N_, heavy isotope-labeled cells treated with DMSO and Noc, respectively. See text for details. **(B)** The IP products were separated by SDS-PAGE on 10% gels and visualized by Coomassie Blue staining. Arrows indicate the protein bands corresponding to lamins A and C that were excised for MS analysis. **(C)** Representative mass spectra of selected pairs of non-phosphopeptides and phosphopeptides from lamin A (upper panel) and lamin C (lower panel) in the SILAC experiment. Data were taken from the first experiment, condition 1, except for SGAQASS_T_PL_S_PTR of lamin C, which was from the second experiment, condition 1.

The SILAC method, used in conjunction with LC-MS/MS, has been shown to be a powerful approach for analyzing quantitative changes in the proteome of viable cells [24,25]. This approach has also been applied to dissect changes in site-specific phosphorylation of target proteins in cells stimulated by extracellular signals [26]. To gain insight into the quantitative changes in the phosphorylation status of lamin A and C phosphorylation sites during mitosis, we applied the SILAC method and LC-MS/MS to quantify the levels of multiple phosphorylation sites of lamin A and C isolated from HeLa S3 cells at interphase and mitosis. The workflow is depicted in Figure 5A. Briefly, HeLa S3 cells were grown in medium containing light or heavy amino acids for six doubling times. In condition 1, ‘light’ and ‘heavy’ cells were treated for 16 h with Noc (1 μg/mL) and DMSO (vehicle control), respectively; treatments were reversed in condition 2 (DMSO and Noc for light and heavy, respectively). This experimental paradigm allowed us to obtain interphase and mitosis populations from both treatment combinations. Extracts from both sets of cells were mixed at a 1:1 ratio and immunoprecipitated using an anti-lamin A/C antibody. The immunoprecipitated products were then separated by SDS-PAGE, followed by Coomassie Blue staining. The protein staining pattern is shown in Figure 5B. Gel bands corresponding to lamins A and C were excised for in-gel tryptic digestion, and the digestion products were subjected to LC-MS/MS analysis, followed by MS data processing and quantitative analysis using the peptide quantification pipeline (see Methods for details).

Using this strategy, we identified and quantified 65 peptides in isolated lamin A and 61 peptides in isolated lamin C (Additional file 2: Table S1), achieving 64% sequence coverage for lamin A and 66% sequence coverage for lamin C (Additional file 1: Figure S3 and S4). Of these peptides, 55 of 65 (84.6%) of those for lamin A and 47 of 61 (77%) of those for lamin C exhibited an average fold-change (Noc-treated/DMSO control) between 0.8 and 1.2. The mean and median of average fold-changes for all quantified peptides were 1.23 and 1.07 for lamin A and 1.27 and 1.13 for lamin C, respectively (Additional file 2: Table S1), indicating that DMSO-treated and Noc-treated cell pools contained approximately equal amounts of lamin A and C proteins, and that the levels of most detected peptides from lamins A and C were not significantly changed by Noc treatment. Most quantitation results were reproducible in a second biological replicate of the experiment (Additional file 2: Table S1). From Additional file 2: Table S1, however, it is clear that Noc treatment significantly altered the amounts of eight peptides (S^12^GAQASSTp^19^PLSp^22^PTR^25^, S^12^GAQASSTPLSp^22^PTR^25^, S^12^GAQASSTPLSPTR^25^, L^387^RLSp^390^PSp^392^PTSQR^397^, L^387^RLSp^390^PSPTSQR^397^, L^389^SPSPTSQR^397^, S^628^VGGSGGGSp^636^FGDNLVTR^644^, and S^628^VGGSGGGSFGDNLVTR^644^) from lamin A and six peptides (S^12^GAQASSTp^19^PLSp^22^PTR^25^, S^12^GAQASSTPLSp^22^PTR^25^, S^12^GAQASSTPLSP- TR^25^, L^387^RLSp^390^PSp^392^PTSQR^397^, L^387^RLSp^390^PSPTSQR^397^, and L^389^SPSPTSQR^397^) from lamin C in HeLa S3 cells. These results are further summarized in Table 1.

**Table 1:** SILAC-based quantification of tryptic phosphopeptides and their corresponding non-phosphopeptides derived from immunoprecipitated lamins A and C

**Lamin A**			
Start	End	Peptide sequence	Normalized Ratio (Noc/DMSO)
12	25	SGAQASS_T_PL_S_PTR	5.40
12	25	SGAQASSTPL_S_PTR	5.49
12	25	SGAQASSTPLSPTR	0.60
387	397	LRL_S_P_S_PTSQR	2.42
387	397	LRL_S_PSPTSQR	0.43
389	397	LSPSPTSQR	0.76
628	644	SVGGSGGG_S_FGDNLVTR	2.02
628	644	SVGGSGGGSFGDNLVTR	0.84
Lamin C			
Start	End	Peptide sequence	Normalized Ratio (Noc/DMSO)
12	25	SGAQASS_T_PL_S_PTR	5.49
12	25	SGAQASSTPL_S_PTR	3.47
12	25	SGAQASSTPLSPTR	0.57
387	397	LRL_S_P_S_PTSQR	2.27
387	397	LRL_S_PSPTSQR	0.47
389	397	LSPSPTSQR	0.70

This quantitative analysis revealed that in mitotic HeLa S3 cells (i) mono-phosphorylation of S^12^GAQASSTPLSPTR^25^ at Ser-22 and di-phosphorylation of S^12^GAQASSTPLSPTR^25^ at both Thr-19 and Ser-22 were the most prominent phosphorylation events (3.5–5.5 fold increase) on lamins A and C; (ii) di-phosphorylation of L^389^SPSPTSQR^397^ at both Ser-390 and Ser-392 on lamins A and C were increased 2.3–2.4 fold, in association with a parallel 53–57% decrease in mono-phosphorylation of L^389^SPSPTSQR^397^ at Ser-390; and (iii) phosphorylation of S^628^VGGSGGGSFGDNLVTR^644^ at Ser-636 on lamin A was augmented 2.0 fold (Table 1). Representative mass spectra of selected non-phosphopeptide and phosphopeptide pairs from lamins A and C in this SILAC-based experiment are presented in Figure 5C.

## Discussion

In this study, we identified phospho-Thr-19 of lamin A/C as the phosphoepitope recognized by P-STM, a polyclonal antibody originally generated against the regulatory autophosphorylation site of PAK2. This phosphoepitope can be created *in vitro* by CDK1-catalyzed phosphorylation of recombinant GST-lamin C-N1 protein (Figure 2). Moreover, an LC-MS/MS analysis of this *in vitro* phosphorylation product clearly indicated that Thr-19 and Ser-22 are the two prominent sites phosphorylated by CDK1 (Figure 4). Taken together with the demonstration by SILAC-based quantitative MS analysis that the level of Thr-19 phosphorylation on lamin A/C increased >5 fold in mitotic HeLa S3 cells (Figure 5 and Table 1), these observations indicate that CDK1-mediated Thr-19 phosphorylation of lamin A/C is responsible for generating the phosphoepitope recognized by P-STM in mitotic cells.

As noted above, the nuclear lamina depolymerizes as a result of mitosis-specific phosphorylation of nuclear lamins at specific sites [3,4]. The functional roles of some phosphorylation sites of lamin A/C in cell-cycle progression or in certain physiological settings have been investigated. For example, mutation of Thr-19, Ser-22, or Ser-392 (phosphorylation site of CDK1) to Ala on lamin A significantly inhibits lamin A disassembly in mitotic cells, whereas mutation of both Ser-403 and Ser-404 (phosphorylation site of other kinases) to Ala inhibits the transport of mutant lamin A to the nucleus [4]. The major phosphorylated sites of lamin A/C are located on both sides of the α-helical central rod domain, and phosphorylation at these sites is known to interfere with the formation of lamin head-to-tail polymers from lamin dimers [27]. Phosphorylation of Ser-22 and Ser-392 of lamin A by CDK5, which induces the nuclear lamin A dispersion that leads to CDK5-mediated neurotoxicity, has been observed in neuronal cells challenged by neurotoxic stimuli (amyloid-β and glutamate) [7]. This CDK5-mediated nuclear lamin A dispersion and neurotoxicity is significantly diminished by introduction of phosphorylation-resistant mutations (S22A, S392A) in lamins, highlighting CDK5-mediated Ser-22/Ser-392 phosphorylation as a major mechanism for the neuronal death elicited by neurotoxic stimuli [7]. In addition, as first demonstrated by Cenni et al. [28], lamin A N-terminal phosphorylation is associated with myoblast activation, and this phosphorylation is significantly impaired in mature muscle fibers from patients with Emery-Dreifuss muscular dystrophy (EDMD). These authors subsequently demonstrated that (i) Akt activated by insulin phosphorylates lamin A/C at Ser-404 in C2C12 mouse myoblasts, but this phosphorylation cannot be detected in primary cells from an EDMD-2 patient; (ii) Akt phosphorylation at Ser-404 targets the precursor prelamin A for degradation; and (iii) Akt regulates transcription of the gene encoding lamin A (*LMNA*) [8,9,29]. Moreover, specific phosphorylation of Ser-458 of A-type lamins in *LMNA*-associated myopathy patients has been reported, although the role of this phosphorylation remains to be established [30].

To facilitate study of the site-specific alteration of phosphorylation levels on lamins during mitosis or in other physiological settings, research laboratories and biotechnology companies have produced several phosphoepitope-specific antibodies against specific phosphorylation sites of lamins. For instance, Kuga developed monoclonal antibodies against five specific phosphorylation sites (Thr-14, Ser-17, Ser-385, Ser-387, and Ser-401) of lamin B2 and used these antibodies to study dynamic changes at these phospho-sites in lamin B2/B1 throughout the cell cycle [31]. However, only a few phosphoepitope-specific antibodies against lamin A/C, including those against phospho-Ser-22, phospho-Ser-392, phospho-Ser-404 [8], and phospho-Ser-458 [30], have been reported, although the antibody raised against the phospho-Ser-17 of lamin B2 should also be included since it recognizes the homologous residue (phospho-Ser-22) in lamin A/C [31]. Our current study demonstrates that the P-STM antibody represents a useful tool for investigating the Thr-19 phosphorylation of lamin A/C in living cells.

LC-MS/MS analyses of the GST–N1 fusion protein *in vitro*-phosphorylated by CDK1 revealed the presence of phospho-Thr-19 and/or phospho-Ser-22 in two mono-phosphopeptides (SGAQASSTPLSp^22^PTR and SGAQASSTp^19^PLSPTR) and one di-phosphopeptide (SGAQASSTp^19^PLSp^22^PTR)(Figure 4B). Using the same LC-MS/MS conditions to analyze lamin A/C immunoprecipitated from equal mixtures of interphase and mitotic HeLa S3 cell extracts, however, we only detected the phospho-Ser-22-containing peptide, SGAQASSTPLSp^22^PTR, and the phospho-Thr-19/phospho-Ser-22-containing peptide, SGAQASSTp^19^PLSp^22^PTR; the phospho-Thr-19-containing peptide, SGAQASSTp^19^PLSPTR, in the N-terminal region of lamin A/C was not detected (Table 1 and Additional file 2: Table S1). This observation seems to indicate that, in mitotic HeLa S3 cells, mono- and dual-phosphorylation of the peptide S^12^GAQASSTPLSPTR^25^ at Ser-22 and Thr-19/Ser-22, respectively, are the major phosphorylation events that occur in the lamin A/C N-terminal region. The mono-phosphorylation of this peptide at Thr-19 was either too low to be detected by LC-MS/MS or did not occur in mitotic HeLa S3 cells. In this scenario, the di-phosphopeptide SGAQASSTp^19^PLSp^22^PTR may represent the major phosphoepitope recognized by P-STM in mitotic HeLa S3 cells. This interpretation is further supported by the observation that P-STM recognized both the phospho-Thr-19-containing peptide, SGAQASSTp^19^PLSPTR, and the phospho-Thr-19/phospho-Ser-22-containing peptide, SGAQASSTp^19^PLSp^22^PTR, in dot-blot experiments (Figure 3B).

Several lines of evidence suggest that the epitopes recognized by P-STM are not sequence-conserved but may be structure-related. To date, three phosphoproteins recognized by P-STM have been identified: PAK2 [20], lamin A/C [21], and MST3 [[32] and Additional file 1: Figure S5]. Like PAK2, MST3 is also a member of the Ste20 kinase family. An alignment and comparison of the amino acid sequences of the phosphoepitopes in the three proteins, S^398^KRS**T**pMVGTPY (for PAK2), I^174^KRN**T**pFVGTPF (for MST3) and S^12^GAQASS**T**pPLSPTR (for lamin A/C), reveals high homology only between the epitope sequences from PAK2 and MST3; no obvious similarity exists between the phosphoepitope from lamin A/C (S^12^GAQASS**T**pPLSPTR) and those from PAK2 or MST3. The fact that phospho-Thr appears to be the only conserved ‘structure’ between these three epitope sequences would seem to suggest that phospho-Thr is the most critical determinant for generating the P-STM phosphoepitope. However, we have observed that several phosphoproteins containing phospho-Thr, including PAK2- and GSK3-phosphorylated MBP, do not react to P-STM (data not shown). Therefore, the microenvironment surrounding phospho-Thr in a phosphoprotein may play an important role in creating the epitope recognized by P-STM. Some additional techniques, including three-dimensional structure analysis of the three phosphoepitopes described above, may be helpful in analyzing the precise structural requirements for producing the P-STM phosphoepitope.

## Conclusions

The data shown here indicate that P-STM is a valuable tool for identifying phosphoproteins involved in regulating the mitotic process and monitoring the phosphorylation level of phospho-Thr-19 on lamin A/C during cell-cycle progression.

## Methods

### Materials

Nocodazole (Noc) was purchased from Sigma (St. Louis, MO, USA). Anti-lamin A/C antibody (N-18), anti-lamin antibody conjugated with agarose (636 AC), and secondary antibodies conjugated with alkaline phosphatase were obtained from Santa Cruz Biotechnology (Santa Cruz, CA, USA). BCA protein assay reagent was from Pierce (Rockford, IL, USA). CDK1 was from Upstate Biotechnology (Lake Placid, NY, USA). Polyvinylidene fluoride (PVDF) membranes were purchased from Millipore (Bedford, MA, USA). CDP-Star (a chemiluminescent substrate for alkaline phosphatase) was from Applied Biosystems (Bedford, MA, USA). P-STM-11-C peptide (SKRSTp^19^MVGTPYC) and peptides A (SGAQASSTp^19^PLSp^22^PTR), B (SGAQASSTp^19^PLSPTR), C (SGAQASSTPLSp^22^PTR), and D (SGAQASSTPLSPTR) were synthesized by Kelowna (Taipei, Taiwan). Sequencing grade trypsin was from Promega (Madison, WI, USA). Synergi Polar-RP 4 μm resin was obtained from Phenomenex (Torrance, CA, USA). Agilent Zorbax 300SB-C18 was from Agilent Technologies (Santa Clara, CA, USA). BioBasic C18 300 Å Packed PicoFrit Columns (PFC7515-BI-10) were from Thermo Fisher Scientific **(**Waltham, MA, USA**)**.

### P-STM antibody production

The P-STM-11-C peptide (SKRSTp^402^MVGTPY-C), a phosphopeptide containing the regulatory autophosphorylation site sequence of human PAK2, was coupled to keyhole limpet hemocyanin and used as an antigen to produce P-STM antibody in rabbits. Purification and characterization of this phospho-specific antibody were as previously described [20].

### Cell culture and stable isotope labeling by amino acids in cell culture (SILAC)

HeLa S3 cells were cultured in Dulbecco’s modified Eagle’s medium (DMEM) supplemented with 10% heat-inactivated fetal bovine serum, 100 units/mL of penicillin/streptomycin (Invitrogen, Carlsbad, CA, USA) at 37°C in a water-saturated 5% CO_2_ atmosphere. The SILAC protocol was essentially as previously described [33]. Briefly, two populations of cells were maintained in lysine- and arginine-depleted DMEM (Biological Industries, Israel) supplemented with 10% heat-inactivated fetal bovine serum, 100 units/mL of penicillin/streptomycin, and either light L-lysine (146 mg/L) and L-arginine (84 mg/L) or heavy [^13^C_6_]L-lysine (146 mg/L) and [^13^C_6_]L-arginine (84 mg/L) (Invitrogen), and additional L-proline (100 mg/L) [34]. Cells were grown for at least six doublings to allow full incorporation of labeled amino acids.

### Drug treatments and cell extract preparation

Cells were arrested at G_2_/M phase by incubating for 16 h with 1 μg/mL nocodazole (Noc), dissolved in dimethyl sulfoxide (DMSO). Cells were then were washed twice with ice-cold phosphate-buffered saline (PBS), and lysed in lysis buffer (20 mM Tris–HCl pH 7.0, 1 mM EDTA, 1 mM EGTA, 1% Triton X-100, 1 mM benzamidine, 1 mM phenylmethylsulfonyl fluoride, 50 mM NaF, 20 mM sodium pyrophosphate, and 1 mM sodium orthovanadate) on ice for 15 min. The cell lysates were collected and sonicated on ice (Model W-380 sonicator; Heat Systems-Ultrasonics Inc.) (Salem, Massachusetts, USA) for 3 × 10 s at 50% power, followed by centrifugation at 50,000 × g for 20 min at 4°C. The supernatant was collected as total cell extract.

### Immunoprecipitation

For routine immunoprecipitation, total cell extracts (1 mg of protein in 0.5 mL of lysis buffer) were incubated with 15 μL of agarose-conjugated anti-lamin A/C antibody (7.5 μg antibody) at 4°C for 12 h with shaking. Immune complexes were collected by centrifugation and washed twice with 0.5 mL of ice-cold solution A (20 mM Tris–HCl pH 7.0 and 0.5 mM dithiothreitol [DTT]) containing 0.5 M NaCl and twice with ice-cold solution A without NaCl. For SILAC experiments, total cell extracts from the two populations of cells were mixed in a 1:1 ratio and then immunoprecipitated as described above.

### Construction of plasmids and expression of fusion proteins

The cDNA sequence for human lamin A/C was retrieved from the National Center for Biotechnology Information (NCBI; GeneBank accession number, X03445). Using the indicated primer pairs and cDNA from A431 cells as a template, the following lamin regions were amplified by polymerase chain reaction (PCR): lamin C-N region (aa 1–375), 5′-CGG GAT CCC GAT GGA GAC CCC GTC CCA G-3′ (forward) and 5′-CGG GAT CCC GGG CGT GGA TCT CCA TGT CC-3′ (reverse); lamin C-N1 region (aa 1–57), 5′-CGG GAT CCC GAT GGA GAC CCC GTC CCA G-3′ (forward) and 5′-CGG AAT TCC TGC GTT CTC CGT TTC CAG C-3′ (reverse); lamin C-N2 region (aa 58–375), 5′-CGG AAT TCC GGG CTG CGC CTT CGC ATC-3′ (forward) and 5′-CGG GAT CCC GGG CGT GGA TCT CCA TGT CC-3′ (reverse); and lamin C-C region (aa 376–572), 5′-CGG GAT CCC GGT ACC GCA AGC TCT TGG AGG-3′ (forward) and 5′-CGG GAT CCC GTC AGC GGC GGC TAC CAC T-3′ (reverse). Expression plasmids for producing GST-fused fragments of lamin C regions were produced by digesting PCR products at *Bam*HI/*Eco*RI restriction sites and then cloning into a *Bam*HI/*Eco*RI-digested pGEX-3× bacterial expression vector (GE Healthcare, Little Chalfont, Buckinghamshire, United Kingdom).

GST-fusion proteins were produced by first transforming *Escherichia coli* BL21 with individual plasmids and growing cells in LB medium containing 100 μg/mL ampicillin. After cells had reached an optical density at 600 nm (OD_600_) of 0.6–0.8, protein expression was induced by adding isopropyl β-D-1-thiogalactopyranoside (IPTG; Sigma) to a final concentration of 1 mM and culturing at 30°C for 4 h. Bacteria were harvested by centrifugation and resuspended in 2 mL of extraction buffer (1 M Tris–HCl pH 7.4, 2.5 M NaCl, 20% Tween 20, and 1 M DTT). Cells were lysed by sonicating suspensions (10-s bursts for 30 min) with a model W-380 sonicator (Heat Systems-Ultrasonics), and GST-fusion proteins were purified using glutathione Sepharose beads (Amersham Biosciences, Sunnyvale, CA, USA).

### Site-directed mutagenesis

All lamin C mutants were prepared by site-directed mutagenesis using the QuikChange mutagenesis method (Stratagene, San Diego, CA, USA) as described by the manufacturer. The following mutants were generated by PCR using the indicated primers and the parent plasmid pGEX-3×-lamin C-N1 (aa 1–57) as a template: Thr-19 to Ala-19, 5′-CGG GAT CCC GAT GGA GAC CCC GTC CCA GCG GCG CGC CAC CCG CAG CGG GGC GCA GGC CAG CTC CGC TCC GCT GTC GCC CAC CCG C-3′; Ser-22 to Ala-22, 5′-CGG GAT CCC GAT GGA GAC CCC GTC CCA GCG GCG CGC CAC CCG CAG CGG GGC GCA GGC CAG CTC CAC TCC GCT GGC GCC CAC CCG CAT CAC CCG G-3′; and Thr-19/Ser-22 to Ala-19/Ala-22, 5′-CGG GAT CCC GAT GGA GAC CCC GTC CCA GCG GCG CGC CAC CCG CAG CGG GGC GCA GGC CAG CTC CGC TCC GCT GGC GCC CAC CCG CAT CAC CCG G-3′. PCR products were digested with the methylation-sensitive restriction endonuclease *Dpn*1 to eliminate the parent plasmid, and the reaction products were transformed into DH5α *E. coli* (Stratagene). All mutants were confirmed by sequencing.

### *In vitro* phosphorylation

Purified GST-lamin C fusion proteins (6 μg each) were phosphorylated *in vitro* by incubation in 30 μL of kinase reaction buffer (20 mM Tris–HCl, 0.5 mM DTT, 20 mM Mg^2+^ and 0.2 mM ATP or [γ-^32^P]ATP) containing 60 ng of CDK1 (Upstate Biotechnology) at 30°C for 2 h. The reaction products were resolved by SDS-PAGE, followed by autoradiography or Western blot analysis. For LC-MS/MS analysis, a portion of the reaction product was reduced (10 mM DTT at 56°C for 1 h), alkylated (30 mM iodoacetamide at room temperature for 30 min in the dark), and trypsin digested (protein/enzyme mass ratio 50:1 at 37°C for 16 h) in 25 mM NH_4_HCO_3_.

### Immunoblotting

Immunoblotting was carried out essentially as previously described [20,21]. Affinity-purified P-STM antibody (1 μg/mL) or commercial anti-lamin A/C antibody (1 μg/mL) was used to probe proteins transferred from SDS gels to PVDF membranes. The proteins of interest were detected using goat anti-rabbit IgG antibody conjugated with alkaline phosphatase and the alkaline phosphatase substrate CDP-Star according to the procedure provided by the manufacturer. For reprobing, membranes were stripped with 2% SDS, 100 mM 2-mercaptoethanol and 62.5 mM Tris at 56°C for 45 min with occasional agitation, washed three times in TTBS buffer (20 mM Tris–HCl at pH 7.4, 0.5 M NaCl and 0.05% [v/v] Tween 20), and then reprobed with another antibody.

### In-gel digestion of proteins and mass spectrometric analysis

Coomassie Blue-stained protein bands were excised from the gel and in-gel digested with trypsin according to a protocol described previously [35]. Briefly, the excised protein bands were washed twice with 50% acetonitrile containing 25 mM NH_4_HCO_3_ for 15 min and then with acetonitrile several times. After drying, the gel pieces were subjected to reduction and alkylation by DTT/iodoacetamide in 25 mM NH_4_HCO_3_, followed by in-gel digestion with freshly prepared enzyme solution (20 ng/μL of trypsin in 25 mM NH_4_HCO_3_) at 37°C for 16 h. The resulting tryptic peptides were reconstituted in HPLC (high-performance liquid chromatography) buffer A (0.05% formic acid; Sigma) and then analyzed by LC-MS/MS. LC-MS/MS data were acquired on a nanoLC U3000 system (Dionex, Sunnyvale, CA, USA) equipped with micrOTOFq (Bruker Daltonik GmbH, Bremen, Germany) and operated using micrOTOF control software. Samples were loaded onto a trap column (Zorbax 300SB-C18, 0.3 × 5 mm; Agilent Technologies, Wilmington, DE, USA) at a flow rate of 5 μL/min in HPLC buffer A. After reducing the flow rate to 25 nL/min using a splitter, peptides were separated on an analytical column (Synergy Hydro-RP capillary RP18 column, 2.5 μm, 0.15 × 100 mm; packed in house) using a gradient of 5% to 80% HPLC buffer B (0.05% formic acid in 100% acetonitrile) in 75 min. Peptide fragment spectra were acquired from one MS scan, followed by four MS/MS scans of the most abundant parent ions. Each precursor was analyzed twice and then excluded in the following minute. MS data were processed with DataAnalysis v.3.4 (Bruker Daltonics) to generate peak lists and .mgf files (Mascot generic format files) for database searches.

MS spectra were searched against the SwissProt-human_56.1 FASTA Database (Homo sapiens, 15720 sequences), assuming trypsin as the digesting enzyme. The MASCOT search engine (http://www.matrixscience.com) (v.2.2.03 Matrix Science, London, UK) was used with the following parameters: one missing cleavage site allowed with charge states from 1^+^ to 3^+^; MS mass tolerance set to 50 ppm and MS/MS tolerance set to 0.1 Da for both fixed modification (carbamidomethylation of Cys) and variable modifications (oxidation of Met, phosphorylation of Ser/Thr, and ^13^C_6_-incorporation of Lys/Arg for SILAC labeling).

### Peptide quantification

Peptide pairs from SILAC samples were quantified as follows. First, peptides with an ion score >15 were identified from the exported MASCOT search results (*.xtmL) using BioTools software (Bruker Daltonics). The search results of each identified phosphopeptide were further checked and verified by manual alignment. Second, the light and heavy peptide pairs were identified using DataAnalysis software (Bruker Daltonics). The retention time (RT) and mass of a light peptide detected in an LC-run were used as bases to set a time window (RT ± 15 s) and a mass window [(mass + 6.02/12.04 Da) ± 0.05 Da)] to find its coeluted, paired heavy peptide. Third, the area of extracted ion chromatogram (EIC) of each light or heavy peptide was obtained and used to calculate the EIC ratio (heavy/light) of each peptide pair using WARP-LC software (Bruker Daltonics). Fourth, for a specific peptide pair with multiple EIC ratios (because of repeated detection in a single LC-run), the mean EIC ratio was calculated and used as the average EIC ratio of each peptide pair detected/quantified in an LC-run. Finally, the quantified results [Noc/Ctrl ratios obtained from EIC ratios (heavy/light)] of each peptide pair from the four LC-runs were averaged to obtain the final average Noc/Ctrl ratio of each peptide pair.

## Abbreviations

CDK: Cyclin-dependent kinase; DMSO: Dimethyl sulfoxide; LC-MS/MS: Liquid chromatography coupled with tandem mass spectrometry; Noc: Nocodazole; PAK: p21-activated kinase; SILAC: Stable isotope labeling by amino acids in cell culture; GST: Glutathione S-transferase.

## Competing interests

The authors declare that they have no competing interests.

## Authors’ contributions

Jeng-Ting Chen designed the experiments and wrote the manuscript; Chia-Wen Ho carried out the molecular genetic studies; Chia-Wen Ho and Ya-Ju Hsieh were responsible for *in vitro* phosphorylation assays; Jeng-Ting Chen, Lang-Ming Chi, and Kun-Yi Chien performed mass spectrometry-based proteome analyses; Jeng-Ting Chen and Shih-Jie Lin carried out the immunoassays; Jau-Song Yu participated in study design and coordination and helped to draft the manuscript. All authors read and approved the final manuscript.

## Supplementary Material

Additional file 1: Figure S1 Assignments of MS/MS spectra of the four synthetic peptides. **Figure S2.** Assignments of MS/MS spectra of the four tryptic peptides obtained from the *in vitro*-phosphorylated sample. **Figure S3.** Sequence coverage of lamin A immunoprecipitated from Noc-treated HeLa S3 cells, as assessed by LC-MS/MS. **Figure S4.** Sequence coverage of lamin C immunoprecipitated from Noc-treated HeLa S3 cells, as assessed by LC-MS/MS. **Figure S5.** Phosphorylation of the regulatory autophosphorylation site Thr-178 of MST3 creates a recognition motif for the P-STM antibody. Click here for file

Additional file 2: Table S1 SILAC-based quantification of tryptic peptides of lamins A and C immunoprecipitated from HeLa S3 cells. Click here for file
