# Supplementary material for: Identification of the lamin A/C phosphoepitope recognized by the antibody P-STM in mitotic HeLa S3 cells
Source: BMC Biochem. 2013 Jul 19;14:18. doi: 10.1186/1471-2091-14-18 (PMC3727946; doi:10.1186/1471-2091-14-18)
Supplement: Additional file 1: Figure S1 — Assignments of MS/MS spectra of the four synthetic peptides. Figure S2. Assignments of MS/MS spectra of the four tryptic peptides obtained from the in vitro-phosphorylated sample. Figure S3. Sequence coverage of lamin A immunoprecipitated from Noc-treated HeLa S3 cells, as assessed by LC-MS/MS. Figure S4. Sequence coverage of lamin C immunoprecipitated from Noc-treated HeLa S3 cells, as assessed by LC-MS/MS. Figure S5. Phosphorylation of the regulatory autophosphorylation site Thr-178 of MST3 creates a recognition motif for the P-STM antibody. [file 1471-2091-14-18-S1.pdf]

Supplemental Fig. 1

Peptide D from Fig. 4A

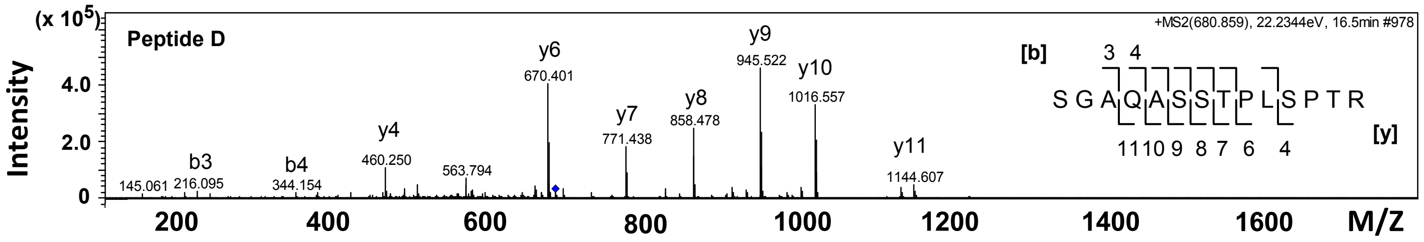

B	B Ions	B + 2H	B - NH_3	B H_2O	B - Neutral loss	AA	Y Ions	Y + 2H	Y -NH_3	Y - H_2O	Y - Neutral loss	Y
1	88.0393	44.5233	71.0128	70.0287		S	1359.6863	680.3468	1342.6598	1341.6757		14
2	145.0608	73.0340	128.0343	127.0502		G	1272.6542	636.8308	1255.6277	1254.6436		13
3	216.0979	108.5526	199.0714	198.0873		A	1215.6328	608.3200	1198.6063	1197.6222		12
4	344.1565	172.5819	327.1300	326.1459		Q	1144.5957	572.8015	1127.5692	1126.5851		11
5	415.1936	208.1004	398.1671	397.1830		A	1016.5371	508.7722	999.5106	998.5265		10
6	502.2256	251.6164	485.1991	484.2150		S	945.5000	473.2536	928.4735	927.4894		9
7	589.2576	295.1325	572.2311	571.2470		S	858.4680	429.7376	841.4415	840.4574		8
8	690.3053	345.6563	673.2788	672.2947		T	771.4359	386.2216	754.4094	753.4253		7
9	787.3581	394.1827	770.3316	769.3475		P	670.3882	335.6978	653.3617	652.3776		6
10	900.4421	450.7247	883.4156	882.4315		L	573.3355	287.1714	556.3090	555.3249		5
11	987.4742	494.2407	970.4477	969.4636		S	460.2514	230.6294	443.2249	442.2408		4
12	1084.5269	542.7671	1067.5004	1066.5163		P	373.2194	187.1133	356.1929	355.2088		3
13	1185.5746	593.2909	1168.5481	1167.5640		T	276.1666	138.5870	259.1401	258.1560		2
14	1359.6863	680.3468	1342.6598	1341.6757		R	175.1190	88.0631	158.0925	157.1084		1

Peptide B from Fig. 4A

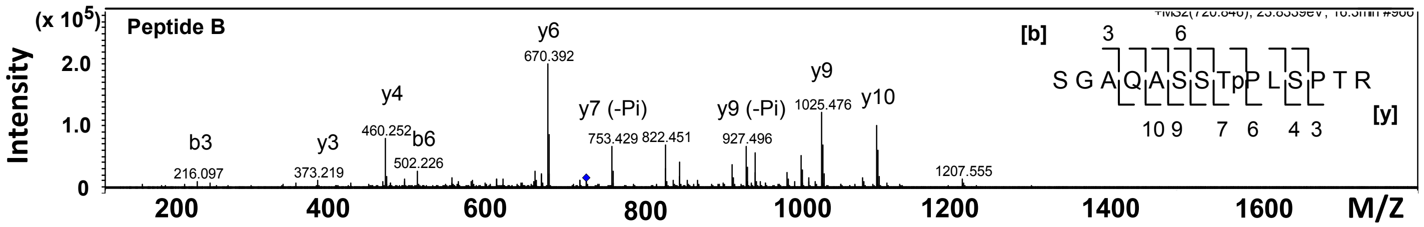

B	B Ions	B + 2H	B - NH_3	B - H_2O	B - Neutral loss	AA	Y Ions	Y + 2H	Y -NH_3	Y - H_2O	Y - Neutral loss	Y
1	88.0393	44.5233	71.0128	70.0287		S	1439.6526	720.3299	1422.6261	1421.6420	1341.6757	14
2	145.0608	73.0340	128.0343	127.0502		G	1352.6206	676.8139	1335.5941	1334.6100	1254.6437	13
3	216.0979	108.5526	199.0714	198.0873		A	1295.5991	648.3032	1278.5726	1277.5885	1197.6222	12
4	344.1565	172.5819	327.1300	326.1459		Q	1224.5620	612.7846	1207.5355	1206.5514	1126.5851	11
5	415.1936	208.1004	398.1671	397.1830		A	1096.5034	548.7554	1079.4769	1078.4928	998.5265	10
6	502.2256	251.6164	485.1991	484.2150		S	1025.4663	513.2368	1008.4398	1007.4557	927.4894	9
7	589.2576	295.1325	572.2311	571.2470		S	938.4343	469.7208	921.4078	920.4237	840.4574	8
8	770.2716	385.6395	753.2451	752.2610	672.2948	Tp	851.4023	426.2048	834.3758	833.3917	753.4254	7
9	867.3244	434.1658	850.2979	849.3138	769.3475	P	670.3882	335.6978	653.3617	652.3776		6
10	980.4085	490.7079	963.3820	962.3979	882.4316	L	573.3355	287.1714	556.3090	555.3249		5
11	1067.4405	534.2239	1050.4140	1049.4299	969.4636	S	460.2514	230.6294	443.2249	442.2408		4
12	1164.4933	582.7503	1147.4668	1146.4827	1066.5164	P	373.2194	187.1133	356.1929	355.2088		3
13	1265.5409	633.2741	1248.5144	1247.5303	1167.5640	T	276.1666	138.5870	259.1401	258.1560		2
14	1439.6526	720.3299	1422.6261	1421.6420	1341.6757	R	175.1190	88.0631	158.0925	157.1084		1

Supplemental Fig. 1. Continued

Peptide C from Fig 4A

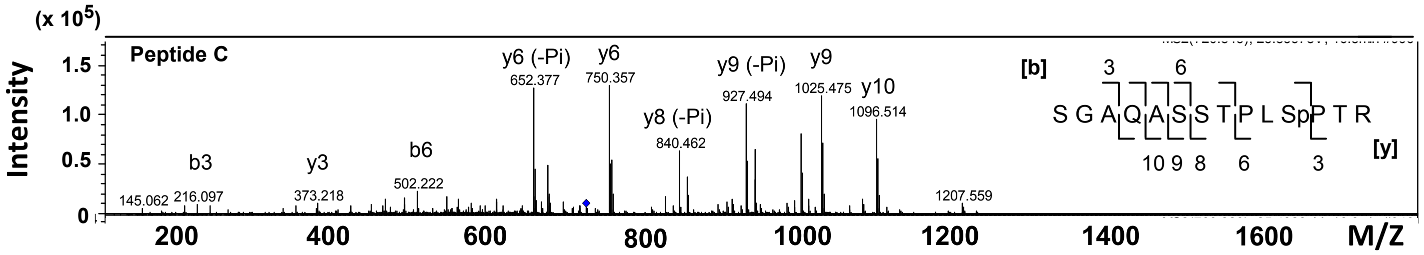

B	B Ions	B + 2H	B - NH_3	B - H_2O	B - Neutral loss	AA	Y Ions	Y + 2H	Y -NH_3	Y - H_2O	Y - Neutral loss	Y
1	88.0393	44.5233	71.0128	70.0287		S	1439.6526	720.3299	1422.6261	1421.6420	1341.6757	14
2	145.0608	73.0340	128.0343	127.0502		G	1352.6206	676.8139	1335.5941	1334.6100	1254.6437	13
3	216.0979	108.5526	199.0714	198.0873		A	1295.5991	648.3032	1278.5726	1277.5885	1197.6222	12
4	344.1565	172.5819	327.1300	326.1459		Q	1224.5620	612.7846	1207.5355	1206.5514	1126.5851	11
5	415.1936	208.1004	398.1671	397.1830		A	1096.5034	548.7554	1079.4769	1078.4928	998.5265	10
6	502.2256	251.6164	485.1991	484.2150		S	1025.4663	513.2368	1008.4398	1007.4557	927.4894	9
7	589.2576	295.1325	572.2311	571.2470		S	938.4343	469.7208	921.4078	920.4237	840.4574	8
8	690.3053	345.6563	673.2788	672.2947		T	851.4023	426.2048	834.3758	833.3917	753.4254	7
9	787.3581	394.1827	770.3316	769.3475		P	750.3546	375.6809	733.3281	732.3440	652.3777	6
10	900.4421	450.7247	883.4156	882.4315		L	653.3018	327.1545	636.2753	635.2912	555.3249	5
11	1067.4405	534.2239	1050.4140	1049.4299	969.4636	Sp	540.2178	270.6125	523.1913	522.2072	442.2409	4
12	1164.4933	582.7503	1147.4668	1146.4827	1066.5164	P	373.2194	187.1133	356.1929	355.2088		3
13	1265.5409	633.2741	1248.5144	1247.5303	1167.5640	T	276.1666	138.5870	259.1401	258.1560		2
14	1439.6526	720.3299	1422.6261	1421.6420	1341.6757	R	175.1190	88.0631	158.0925	157.1084		1

Peptide A from Fig 4A

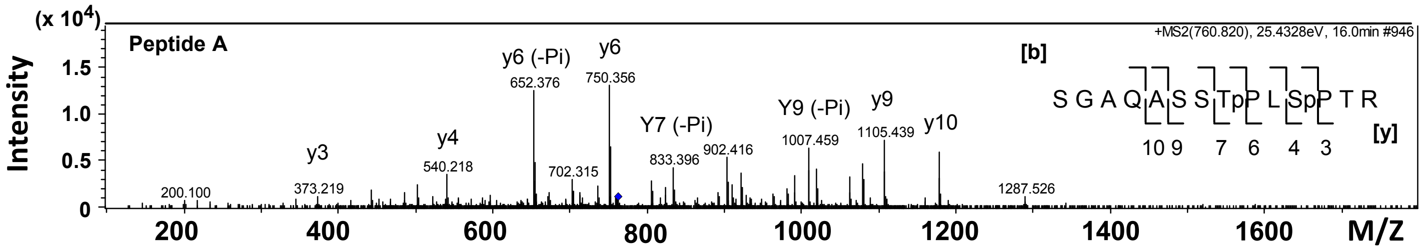

B	B Ions	B + 2H	B - NH_3	B - H_2O	B - Neutral loss	AA	Y Ions	Y + 2H	Y -NH_3	Y - H_2O	Y - Neutral loss	Y
1	88.0393	44.5233	71.0128	70.0287		S	1519.6189	760.3131	1502.5924	1501.6083	1421.6420	14
2	145.0608	73.0340	128.0343	127.0502		G	1432.5869	716.7971	1415.5604	1414.5763	1334.6100	13
3	216.0979	108.5526	199.0714	198.0873		A	1375.5654	688.2864	1358.5389	1357.5548	1277.5886	12
4	344.1565	172.5819	327.1300	326.1459		Q	1304.5283	652.7678	1287.5018	1286.5177	1206.5514	11
5	415.1936	208.1004	398.1671	397.1830		A	1176.4698	588.7385	1159.4433	1158.4592	1078.4929	10
6	502.2256	251.6164	485.1991	484.2150		S	1105.4326	553.2200	1088.4061	1087.4220	1007.4558	9
7	589.2576	295.1325	572.2311	571.2470		S	1018.4006	509.7039	1001.3741	1000.3900	920.4237	8
8	770.2716	385.6395	753.2451	752.2610	672.2948	Tp	931.3686	466.1879	914.3421	913.3580	833.3917	7
9	867.3244	434.1658	850.2979	849.3138	769.3475	P	750.3546	375.6809	733.3281	732.3440	652.3777	6
10	980.4085	490.7079	963.3820	962.3979	882.4316	L	653.3018	327.1545	636.2753	635.2912	555.3249	5
11	1147.4068	574.2071	1130.3803	1129.3962	1049.4299	Sp	540.2178	270.6125	523.1913	522.2072	442.2409	4
12	1244.4596	622.7334	1227.4331	1226.4490	1146.4827	P	373.2194	187.1133	356.1929	355.2088		3
13	1345.5073	673.2573	1328.4808	1327.4967	1247.5304	T	276.1666	138.5870	259.1401	258.1560		2
14	1519.6189	760.3131	1502.5924	1501.6083	1421.6420	R	175.1190	88.0631	158.0925	157.1084		1

Supplemental Fig. 2

Peptide D from Fig. 4B

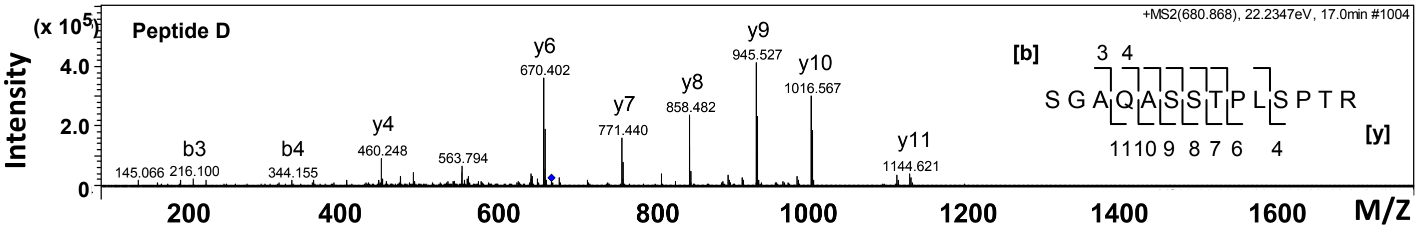

B	B Ions	B + 2H	B - NH_3	B H_2O	B - Neutral loss	AA	Y Ions	Y + 2H	Y -NH_3	Y - H_2O	Y - Neutral loss	Y
1	88.0393	44.5233	71.0128	70.0287		S	1359.6863	680.3468	1342.6598	1341.6757		14
2	145.0608	73.0340	128.0343	127.0502		G	1272.6542	636.8308	1255.6277	1254.6436		13
3	**216.0979**	108.5526	199.0714	198.0873		A	1215.6328	608.3200	1198.6063	1197.6222		12
4	**344.1565**	172.5819	327.1300	326.1459		Q	**1144.5957**	572.8015	1127.5692	1126.5851		11
5	415.1936	208.1004	398.1671	397.1830		A	**1016.5371**	508.7722	999.5106	998.5265		10
6	502.2256	251.6164	485.1991	484.2150		S	**945.5000**	473.2536	928.4735	927.4894		9
7	589.2576	295.1325	572.2311	571.2470		S	**858.4680**	429.7376	841.4415	840.4574		8
8	690.3053	345.6563	673.2788	672.2947		T	**771.4359**	386.2216	754.4094	753.4253		7
9	787.3581	394.1827	770.3316	769.3475		P	**670.3882**	335.6978	653.3617	652.3776		6
10	900.4421	450.7247	883.4156	882.4315		L	573.3355	287.1714	556.3090	555.3249		5
11	987.4742	494.2407	970.4477	969.4636		S	**460.2514**	230.6294	443.2249	442.2408		4
12	1084.5269	542.7671	1067.5004	1066.5163		P	373.2194	187.1133	356.1929	355.2088		3
13	1185.5746	593.2909	1168.5481	1167.5640		T	276.1666	138.5870	259.1401	258.1560		2
14	1359.6863	680.3468	1342.6598	1341.6757		R	175.1190	88.0631	158.0925	157.1084		1

Peptide B from Fig. 4B

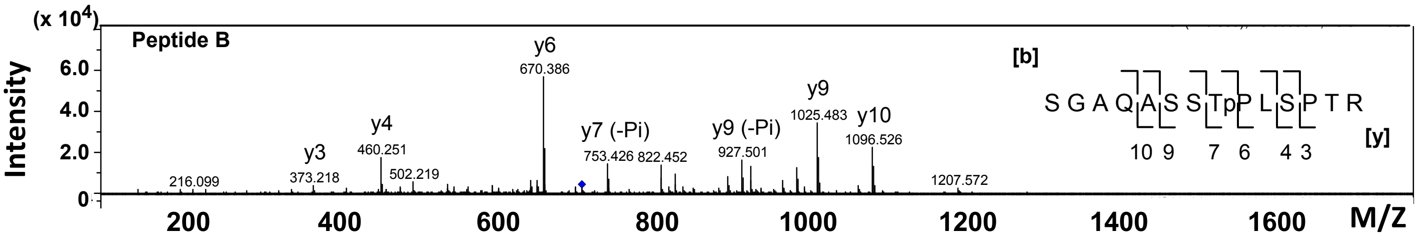

B	B Ions	B + 2H	B - NH_3	B - H_2O	B - Neutral loss	AA	Y Ions	Y + 2H	Y -NH_3	Y - H_2O	Y - Neutral loss	Y
1	88.0393	44.5233	71.0128	70.0287		S	1439.6526	720.3299	1422.6261	1421.6420	1341.6757	14
2	145.0608	73.0340	128.0343	127.0502		G	1352.6206	676.8139	1335.5941	1334.6100	1254.6437	13
3	216.0979	108.5526	199.0714	198.0873		A	1295.5991	648.3032	1278.5726	1277.5885	1197.6222	12
4	344.1565	172.5819	327.1300	326.1459		Q	1224.5620	612.7846	1207.5355	1206.5514	1126.5851	11
5	415.1936	208.1004	398.1671	397.1830		A	**1096.5034**	548.7554	1079.4769	1078.4928	998.5265	10
6	502.2256	251.6164	485.1991	484.2150		S	**1025.4663**	513.2368	1008.4398	1007.4557	**927.4894**	9
7	589.2576	295.1325	572.2311	571.2470		S	938.4343	469.7208	921.4078	920.4237	840.4574	8
8	770.2716	385.6395	753.2451	752.2610	672.2948	Tp	851.4023	426.2048	834.3758	833.3917	**753.4254**	7
9	867.3244	434.1658	850.2979	849.3138	769.3475	P	**670.3882**	335.6978	653.3617	652.3776		6
10	980.4085	490.7079	963.3820	962.3979	882.4316	L	573.3355	287.1714	556.3090	555.3249		5
11	1067.4405	534.2239	1050.4140	1049.4299	969.4636	S	**460.2514**	230.6294	443.2249	442.2408		4
12	1164.4933	582.7503	1147.4668	1146.4827	1066.5164	P	**373.2194**	187.1133	356.1929	355.2088		3
13	1265.5409	633.2741	1248.5144	1247.5303	1167.5640	T	276.1666	138.5870	259.1401	258.1560		2
14	1439.6526	720.3299	1422.6261	1421.6420	1341.6757	R	175.1190	88.0631	158.0925	157.1084		1

Supplemental Fig. 2. Continued

Peptide C from Fig. 4B

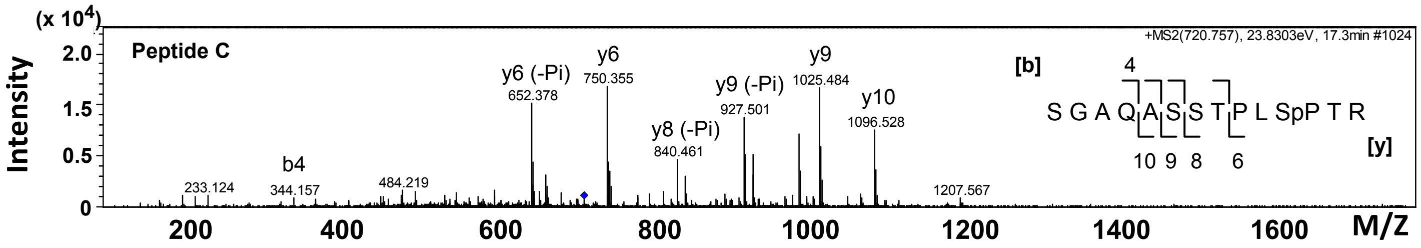

B	B Ions	B + 2H	B - NH_3	B - H_2O	B - Neutral loss	AA	Y Ions	Y + 2H	Y -NH_3	Y - H_2O	Y - Neutral loss	Y
1	88.0393	44.5233	71.0128	70.0287		S	1439.6526	720.3299	1422.6261	1421.6420	1341.6757	14
2	145.0608	73.0340	128.0343	127.0502		G	1352.6206	676.8139	1335.5941	1334.6100	1254.6437	13
3	216.0979	108.5526	199.0714	198.0873		A	1295.5991	648.3032	1278.5726	1277.5885	1197.6222	12
4	**344.1565**	172.5819	327.1300	326.1459		Q	1224.5620	612.7846	1207.5355	1206.5514	1126.5851	11
5	415.1936	208.1004	398.1671	397.1830		A	**1096.5034**	548.7554	1079.4769	1078.4928	998.5265	10
6	502.2256	251.6164	485.1991	484.2150		S	**1025.4663**	513.2368	1008.4398	1007.4557	**927.4894**	9
7	589.2576	295.1325	572.2311	571.2470		S	938.4343	469.7208	921.4078	920.4237	**840.4574**	8
8	690.3053	345.6563	673.2788	672.2947		T	851.4023	426.2048	834.3758	833.3917	753.4254	7
9	787.3581	394.1827	770.3316	769.3475		P	**750.3546**	375.6809	733.3281	732.3440	**652.3777**	6
10	900.4421	450.7247	883.4156	882.4315		L	653.3018	327.1545	636.2753	635.2912	555.3249	5
11	1067.4405	534.2239	1050.4140	1049.4299	969.4636	Sp	540.2178	270.6125	523.1913	522.2072	442.2409	4
12	1164.4933	582.7503	1147.4668	1146.4827	1066.5164	P	373.2194	187.1133	356.1929	355.2088		3
13	1265.5409	633.2741	1248.5144	1247.5303	1167.5640	T	276.1666	138.5870	259.1401	258.1560		2
14	1439.6526	720.3299	1422.6261	1421.6420	1341.6757	R	175.1190	88.0631	158.0925	157.1084		1

Peptide A from Fig. 4B

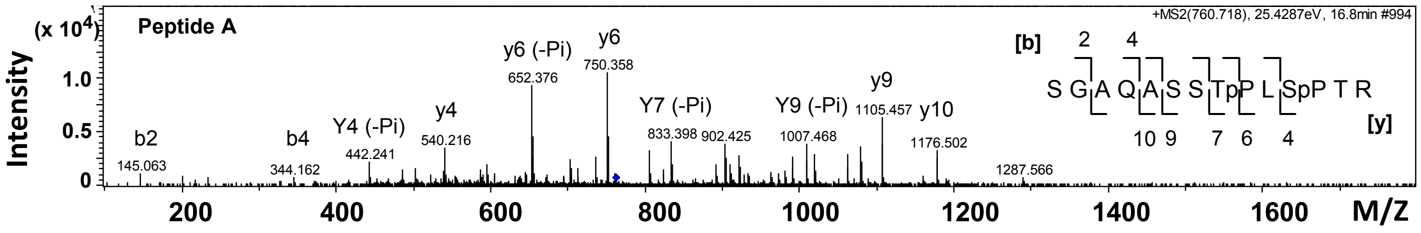

B	B Ions	B + 2H	B - NH_3	B - H_2O	B - Neutral loss	AA	Y Ions	Y + 2H	Y -NH_3	Y - H_2O	Y - Neutral loss	Y
1	88.0393	44.5233	71.0128	70.0287		S	1519.6189	760.3131	1502.5924	1501.6083	1421.6420	14
2	**145.0608**	73.0340	128.0343	127.0502		G	1432.5869	716.7971	1415.5604	1414.5763	1334.6100	13
3	216.0979	108.5526	199.0714	198.0873		A	1375.5654	688.2864	1358.5389	1357.5548	1277.5886	12
4	**344.1565**	172.5819	327.1300	326.1459		Q	1304.5283	652.7678	1287.5018	1286.5177	1206.5514	11
5	415.1936	208.1004	398.1671	397.1830		A	**1176.4698**	588.7385	1159.4433	1158.4592	1078.4929	10
6	502.2256	251.6164	485.1991	484.2150		S	**1105.4326**	553.2200	1088.4061	1087.4220	**1007.4558**	9
7	589.2576	295.1325	572.2311	571.2470		S	1018.4006	509.7039	1001.3741	1000.3900	920.4237	8
8	770.2716	385.6395	753.2451	752.2610	672.2948	Tp	931.3686	466.1879	914.3421	913.3580	**833.3917**	7
9	867.3244	434.1658	850.2979	849.3138	769.3475	P	**750.3546**	375.6809	733.3281	732.3440	**652.3777**	6
10	980.4085	490.7079	963.3820	962.3979	882.4316	L	653.3018	327.1545	636.2753	635.2912	555.3249	5
11	1147.4068	574.2071	1130.3803	1129.3962	1049.4299	Sp	**540.2178**	270.6125	523.1913	522.2072	**442.2409**	4
12	1244.4596	622.7334	1227.4331	1226.4490	1146.4827	P	**373.2194**	187.1133	356.1929	355.2088		3
13	1345.5073	673.2573	1328.4808	1327.4967	1247.5304	T	276.1666	138.5870	259.1401	258.1560		2
14	1519.6189	760.3131	1502.5924	1501.6083	1421.6420	R	175.1190	88.0631	158.0925	157.1084		1

Supplemental Fig. 3

Homo sapiens lamin A/C (LMNA), transcript variant 1, mRNA (NCBI Reference Sequence: NM_170707.2) (Sequence coverage: 64.01%)

```
  1 METPSQRRAT RSGAQASSTP LSPTRITRLQ EKEDLQELND RLAVYIDRVR
 51 SLETENAGLR LRITESEEVV SREVSGIKAA YEAELGDARK TLDSVAKERA
101 RLQLELSKVR EEFKELKARN TKKEGDLIAA QARLKDLEAL LNSKEAALST
151 ALSEKRTLEG ELHDLRGQVA KLEAALGEAK KQLQDEMLRR VDAENRLQTM
201 KEELDFQKNI YSEELRETKR RHETRLVEID NGKQREFESR LADALQELRA
251 QHEDQVEQYK KELEKTYSAK LDNARQSAER NSNLVGAAHE ELQQSRIRID
301 SLSAQLSQLQ KQLAAKEAKL RDLEDSLARE RDTSRRLLAE KEREMAEMRA
351 RMQQQLDEYQ ELLDIKLALD MEIHAYRKLL EGEEERLRLS PSPTSQRSRG
401 RASSHSSQTQ GGGSVTKKRK LESTESRSSF SQHARTSGRV AVEEVDEEGK
451 FVRLRNKSNE DQSMGNWQIK RQNGDDPLLT YRFPPKFTLK AGQVVTIWAA
501 GAGATHSPPT DLVWKAQNTW GCGNSLRTAL INSTGEEVAM RKLVRSVTVV
551 EDDEDEDGDD LLHHHHGSHC SSSGDPAEYN LRSRTVLCGT CGQPADKASA
601 SGSGAQVGGP ISSGSSASSV TVTRSYRSVG GSGGGSFGDN LVTRSYLLGN
651 SSPRTQSPQN CSIM
```

Supplemental Figure 3. Sequence coverage of lamin A immunoprecipitated from nocodazole-treated HeLa S3 cells, as assessed by LC-MS/MS.
Lamin A (664 a.a. residues) immunoprecipitated from nocodazole-treated cells was digested by trypsin and subjected to LC-MS/MS analysis. Four hundred and twenty five residues (425/664, 64.01%) were identified in the peptides shown in Supplemental Table 1 (bold blue) including five phosphorylation sites (bold red), while 239 residues (239/664, 35.99%, black and underlined) were not. The 239 unidentified amino acid residues contain many short peptide sequences with multiple Lys or Arg residues, indicating that we had good sequence coverage for the MS analysis of lamin A.

Supplemental Fig. 4

Isoform C of Prelamin-A/C (LMNA) (Accession Number: LMNA_HUMAN) (Sequence coverage: 66.43%)

1 METPSQRRAT R**SGAQASSTP** **LSPTR**ITR**LQ** **EKEDLQELND** **RLAVYIDR**VR
51 **SLETENAGLR** LR**ITESEEVV** **SR**EVSGIK**AA** **YEAELGDARK** TLDSVAKERA
101 R**LQLELSK**VR EEFKELKARN TK**KEGDLIAA** **QARLKDLEAL** **LNSKEAALST**
151 **ALSEKRTLEG** **ELHDLR**GQVA K**LEAALGEAK** **KQLQDEMLR**R VDAENR**LQTM**
201 **KEELDFQKNI** **YSEELR**ETKR RHETRLVEID NGKQREFESR **LADALQELRA**
251 Q**HEDQVEQYK** **K**ELEKTYSAK LDNARQSAER **NSNLVGAAHE** **ELQQSR**IRID
301 **SLSAQLSQLQ** **K**QLAAKEAK**L** **RDLEDSLAR**E RDTSRRLLAE KEREMAEMRA
351 R**MQQQLDEYQ** **ELLDIKLALD** **MEIHAYRKLL** **EGEEERLRLS** **PSPTSQR**SRG
401 R**ASSHSSQTQ** **GGGSVTK**KR**K** **LESTESRSSF** **SQHAR**TSGR**V** **AVEEVDEEGK**
451 **F**VRLRNK**SNE** **DQSMGNWQIK** **RQNGDDPLLT** **YR**FPPKFTLK **AGQVVTIWAA**
501 **GAGATHSPPT** **DLVWKAQNTW** **GCGNSLRTAL** **INSTGEEVAM** **RK**LVRSVTVV
551 EDDEDEDGDD LLHHHHVSGS RR

Supplemental Figure 4. Sequence coverage of lamin C immunoprecipitated from nocodazole-treated HeLa S3 cells, as assessed by LC-MS/MS.
Lamin C (572 a.a. residues) immunoprecipitated from nocodazole-treated cells was digested by trypsin and subjected to LC-MS/MS analysis. Three hundred and eight residues (380/572, 66.43%) were identified in the peptides shown in Supplemental Table 1 (bold blue), including four phosphorylation sites (bold red), while 192 residues (192/572, 33.57%, black and underlined) were not. The 192 unidentified amino acid residues contain many short peptide sequences with multiple Lys or Arg residues, indicating that we had good sequence coverage for the MS analysis of lamin C.

Supplemental Fig. 5

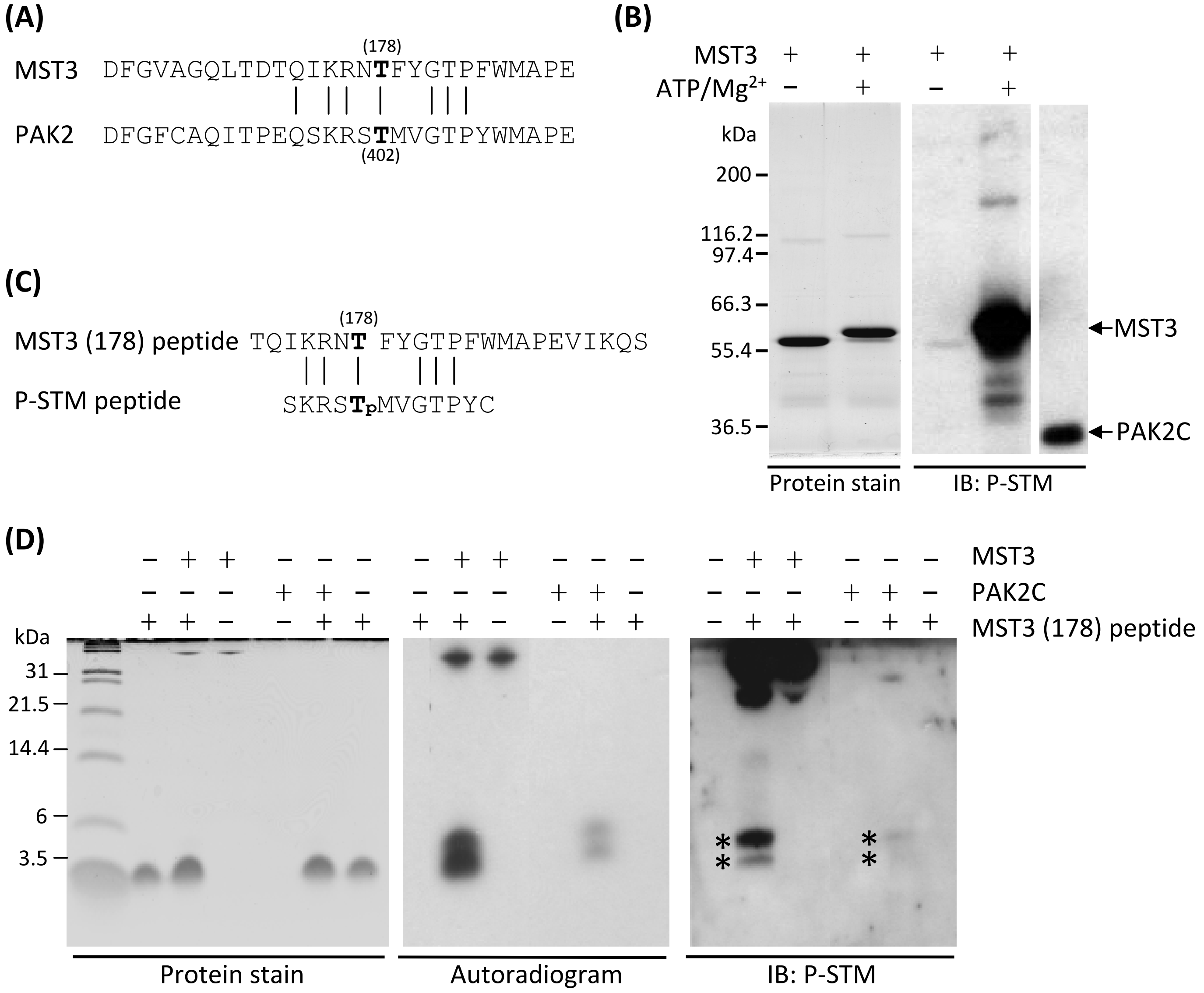

Supplemental Figure 5. Phosphorylation of the regulatory autophosphorylation site Thr-178 of MST3 creates a recognition motif for the P-STM antibody.

(A) Comparison of the regulatory autophosphorylation site sequence between conserved kinase subdomains VII and VIII of MST3 and PAK2. **(B)** Recombinant His-tagged MST3 (4.5 μg), expressed and purified from baculovirus-infected insect cells, was incubated with or without ATP/Mg^{2+} for 16 min and then analyzed by SDS-PAGE on 10% gels followed by Coomassie Blue staining or immunoblotting with the P-STM antibody. The autophosphorylated/activated PAK2 catalytic fragment (PAK2C) was analyzed in parallel as a positive control. **(C)**. The amino acid sequence of a synthetic peptide corresponding to aa 172-193 of MST3 is aligned with the phosphopeptide used to generate P-STM antibody in rabbits. Residues that are identical between the two peptides are denoted by short lines. **(D)** The MST3(178) peptide (25 μg) was incubated in a 25-μl reaction mixture containing 0.2 mM [γ-^{32}P]ATP, 20 mM Mg^{2+}, and recombinant His-tagged MST3 (0.3 μg) or purified PAK2C at 30°C for 30 min. The reaction products were resolved by tricine-SDS-PAGE on a 16.5% gel followed by autoradiography of the dried gel. A portion of the reaction products was resolved in tricine-SDS-PAGE on a 16.5% gel and electrotransferred to PVDF (polyvinylidene difluoride) membrane. The membrane was then probed with the P-STM antibody. Asterisks indicate the phospho-MST3 (178) peptide that was recognized by P-STM.
