# Supplementary material for: Identification of the lamin A/C phosphoepitope recognized by the antibody P-STM in mitotic HeLa S3 cells
Source: BMC Biochem. 2013 Jul 19;14:18. doi: 10.1186/1471-2091-14-18 (PMC3727946; doi:10.1186/1471-2091-14-18)
Supplement: Additional file 2: Table S1 — SILAC-based quantification of tryptic peptides of lamins A and C immunoprecipitated from HeLa S3 cells. [file 1471-2091-14-18-S2.pdf]

Supplemental Table 1. SILAC-based quantification of tryptic peptides of lamins A and C immunoprecipitated from HeLa S3 cells

Lamin A			1st experiment		2nd experiment				
			Condition 1	Condition 2	Condition 1	Condition 2			
Start	End	Peptide sequence	(Light$_{Noc}$/Heavy$_{DMSO}$)	(Heavy$_{Noc}$/Light$_{DMSO}$)	(Light$_{Noc}$/Heavy$_{DMSO}$)	(Heavy$_{Noc}$/Light$_{DMSO}$)	Averaged Ratio (Noc/DMSO)	Mean (Median)	Normalized Ratio (Noc/DMSO)
12	25	SGAQASS$_T$PL$_S$PTR	4.08	7.39	N.D.	5.86	5.77		5.40
12	25	SGAQASSTPL$_S$PTR	6.36	5.40	N.D.	N.D.	5.88		5.49
12	25	SGAQASSTPLSPTR	0.77	0.48	0.77	0.56	0.65		0.60
29	41	LQEKEDLQELNDR	1.01	1.12	0.55	0.90	0.90		0.84
33	41	EDLQELNDR	1.13	0.84	N.D.	0.90	0.96		0.89
42	48	LAVYIDR	1.23	0.98	1.13	N.D.	1.11		1.04
51	60	SLETENAGLR	1.12	1.14	1.23	0.99	1.12		1.05
63	72	ITESEEVVSR	0.69	1.33	0.83	1.37	1.05		0.99
79	89	AAYEAELGDAR	1.40	1.35	1.35	1.01	1.28		1.20
79	90	AAYEAELGDARK	1.04	1.11	0.99	1.10	1.06		0.99
102	108	LQLELSK	1.10	1.08	1.06	1.02	1.07		1.00
123	133	KEGDLIAAQAR	1.18	1.08	N.D.	N.D.	1.13		1.06
124	133	EGDLIAAQAR	1.16	0.88	1.03	1.08	1.04		0.97
134	144	LKDLEALLNSK	0.50	0.89	0.70	1.76	0.96		0.90
136	144	DLEALLNSK	1.09	1.03	1.17	0.93	1.05		0.98
145	155	EAALSTALSEK	0.91	0.75	1.70	1.10	1.11		1.04
145	156	EAALSTALSEKR	0.76	1.32	N.D.	1.07	1.05		0.98
156	166	RTLEGELHDLR	1.15	N.D.	N.D.	N.D.	1.15		1.07
157	166	TLEGELHDLR	0.74	1.03	1.19	0.96	0.98		0.92
172	180	LEAALGEAK	1.08	1.05	1.00	N.D.	1.04		0.97
172	181	LEAALGEAKK	1.12	1.12	N.D.	1.28	1.17		1.10
181	189	KQLQDE$_M$LR	1.05	1.15	N.D.	1.05	1.08		1.01
181	189	KQLQDEMLR	1.32	0.75	1.42	1.05	1.13		1.06
182	189	QLQDE$_M$LR	0.93	0.97	1.04	N.D.	0.98		0.92
182	189	QLQDEMLR	1.13	0.93	1.57	0.98	1.15		1.08
190	196	RVDAENR	N.D.	N.D.	N.D.	0.97	0.97		0.91
197	208	LQT$_M$KEELDFQK	1.02	1.07	1.05	1.03	1.04		0.97
197	208	LQTMKEELDFQK	1.07	1.06	1.35	0.96	1.11		1.04
202	208	EELDFQK	1.25	0.98	N.D.	0.97	1.07		1.00
209	216	NIYSEELR	1.30	0.99	N.D.	1.04	1.11		1.04
226	233	LVEIDNGK	1.24	1.02	N.D.	N.D.	1.13		1.05
241	249	LADALQELR	0.98	1.30	0.66	N.D.	0.98		0.91
250	260	AQHEDQVEQYK	1.08	1.40	1.15	1.02	1.16	1.23 (1.07)	1.08
250	261	AQHEDQVEQYKK	1.04	1.07	0.90	1.27	1.07		1.00
281	296	NSNLVGAAHEELQQSR	0.96	1.24	1.16	1.00	1.09		1.02

297	311	IRIDSLSAQLSQLQK	1.06	1.09	1.37	N.D.	1.18		1.10
299	311	IDSLSAQLSQLQK	1.14	1.21	0.91	0.95	1.05		0.98
320	329	LRDLEDSLAR	1.12	0.99	1.23	0.97	1.08		1.01
322	329	DLEDSLAR	1.21	1.10	N.D.	N.D.	1.16		1.08
342	349	EREMAEMR	1.29	N.D.	N.D.	N.D.	1.29		1.20
352	366	$_{M}$QQQLDEYQELLDIK	1.03	1.11	N.D.	N.D.	1.07		1.00
352	366	MQQQLDEYQELLDIK	1.05	1.16	N.D.	N.D.	1.10		1.03
367	377	LALD$_{M}$EIHAYR	1.13	1.01	1.18	0.97	1.07		1.00
367	377	LALDMEIHAYR	1.14	1.06	N.D.	1.18	1.13		1.05
378	386	KLLEGEEER	1.08	0.94	N.D.	N.D.	1.01		0.94
379	386	LLEGEEER	N.D.	1.00	1.19	1.12	1.10		1.03
387	397	LRL$_{S}$P$_{S}$PTSQR	2.43	2.75	2.41	2.75	2.59		2.42
387	397	LRL$_{S}$PSPTSQR	0.41	0.49	0.49	N.D.	0.46		0.43
389	397	LSPSPTSQR	1.14	0.50	N.D.	N.D.	0.82		0.76
420	427	KLESTESR	N.D.	N.D.	1.11	0.94	1.03		0.96
428	435	SSFSQHAR	N.D.	N.D.	1.48	1.01	1.24		1.16
440	450	VAVEEVDEEGK	1.07	1.04	0.93	1.68	1.18		1.10
440	453	VAVEEVDEEGKFVR	1.05	1.08	1.10	1.09	1.08		1.01
458	470	SNEDQS$_{M}$GNWQIK	0.96	1.09	N.D.	1.11	1.06		0.99
458	470	SNEDQSMGNWQIK	1.05	1.14	1.09	1.27	1.14		1.06
471	482	RQNGDDPLLTYR	N.D.	N.D.	0.90	N.D.	0.90		0.84
472	482	QNGDDPLLTYR	1.16	0.97	N.D.	0.90	1.01		0.94
516	527	AQNTWGCGNSLR	1.12	1.05	1.22	0.96	1.09		1.02
528	541	TALINSTGEEVA$_{M}$R	1.11	N.D.	1.20	0.99	1.10		1.03
528	541	TALINSTGEEVAMR	0.54	0.83	0.95	1.05	0.84		0.79
528	542	TALINSTGEEVA$_{M}$RK	N.D.	N.D.	N.D.	0.54	0.54		0.50
585	597	TVLCGTCGQPADK	0.95	1.07	0.95	1.04	1.00		0.94
598	624	ASASGSGAQVGGPISSGSSASSVTVTR	1.07	1.04	N.D.	N.D.	1.06		0.99
628	644	SVGGSGGG$_{S}$FGDNLVTR	1.89	1.59	4.11	1.03	2.16		2.02
628	644	SVGGSGGGSFGDNLVTR	0.68	1.05	0.82	1.03	0.89		0.84

**Lamin C**			**1st experiment**		**2nd experiment**				
			**Condition 1**	**Condition 2**	**Condition 1**	**Condition 2**			
**Start**	**End**	**Peptide sequence**	**(Light$_{Noc}$/Heavy$_{DMSO}$)**	**(Heavy$_{Noc}$/Light$_{DMSO}$)**	**(Light$_{Noc}$/Heavy$_{DMSO}$)**	**(Heavy$_{Noc}$/Light$_{DMSO}$)**	**Averaged Ratio (Noc/DMSO)**	**Mean (Median)**	**Normalized Ratio (Noc/DMSO)**
12	25	SGAQASS$_{T}$PL$_{S}$PTR	N.D.	N.D.	6.91	5.49	6.20		5.49
12	25	SGAQASSTPL$_{S}$PTR	4.01	3.39	3.95	4.31	3.92		3.47
12	25	SGAQASSTPLSPTR	0.55	0.57	0.64	0.83	0.65		0.57
29	41	LQEKEDLQELNDR	1.03	N.D.	1.26	1.20	1.16		1.03
33	41	EDLQELNDR	1.17	1.07	1.08	N.D.	1.11		0.98

42	48	LAVYIDR	1.13	1.08	1.15	1.24	1.15		1.02
51	60	SLETENAGLR	0.49	1.60	0.93	1.17	1.05		0.93
63	72	ITESEEVVSR	1.00	2.46	1.34	1.42	1.56		1.38
79	89	AAYEAELGDAR	0.92	2.02	1.29	1.41	1.41		1.25
79	90	AAYEAELGDARK	1.00	1.22	0.99	1.29	1.13		1.00
102	108	LQLELSK	N.D.	1.16	1.00	N.D.	1.08		0.96
123	133	KEGDLIAAQAR	1.08	1.13	1.13	1.19	1.13		1.00
124	133	EGDLIAAQAR	1.09	1.06	1.23	N.D.	1.13		1.00
134	144	LKDLEALLNSK	0.40	2.53	0.67	1.05	1.16		1.03
136	144	DLEALLNSK	1.12	1.20	1.10	1.07	1.12		0.99
145	155	EAALSTALSEK	1.16	1.06	1.02	1.02	1.07		0.94
145	156	EAALSTALSEKR	1.00	1.34	1.03	1.24	1.15		1.02
157	166	TLEGELHDLR	0.56	1.54	1.08	1.52	1.17		1.04
172	180	LEAALGEAK	1.04	1.12	N.D.	N.D.	1.08		0.95
172	181	LEAALGEAKK	1.04	1.38	0.88	1.41	1.18		1.04
181	189	KQLQDE$_{M}$LR	N.D.	1.20	1.13	1.19	1.17		1.04
181	189	KQLQDEMLR	1.27	1.04	1.78	N.D.	1.37		1.21
182	189	QLQDE$_{M}$LR	0.89	1.63	N.D.	N.D.	1.26		1.11
182	189	QLQDEMLR	1.09	1.12	N.D.	1.16	1.12		0.99
197	208	LQT$_{M}$KEELDFQK	1.00	0.96	0.99	1.21	1.04		0.92
197	208	LQTMKEELDFQK	1.01	1.25	0.99	1.31	1.14		1.01
202	208	EELDFQK	1.05	1.10	1.01	N.D.	1.06		0.94
209	216	NIYSEELR	1.08	1.15	0.93	1.24	1.10		0.98
241	249	LADALQELR	0.94	1.23	0.87	1.19	1.06		0.94
250	260	AQHEDQVEQYK	1.04	1.07	1.02	1.18	1.08		0.95
250	261	AQHEDQVEQYKK	1.05	1.19	0.79	1.40	1.11	1.27 (1.13)	0.98
281	296	NSNLVGAAHEELQQSR	0.92	1.10	0.96	1.14	1.03		0.91
297	311	IRIDSLSAQLSQLQK	1.03	1.20	N.D.	1.24	1.16		1.02
299	311	IDSLSAQLSQLQK	0.67	1.01	1.00	1.38	1.01		0.90
320	329	LRDLEDSLAR	1.08	1.22	0.95	1.11	1.09		0.97
322	329	DLEDSLAR	1.17	1.11	N.D.	N.D.	1.14		1.01
352	366	$_{M}$QQQLDEYQELLDIK	1.00	1.24	N.D.	N.D.	1.12		0.99
352	366	MQQQLDEYQELLDIK	1.05	1.30	N.D.	N.D.	1.18		1.04
367	377	LALD$_{M}$EIHAYR	N.D.	N.D.	1.06	N.D.	1.06		0.94
367	377	LALDMEIHAYR	1.05	1.16	N.D.	N.D.	1.11		0.98
367	378	LALDMEIHAYRK	0.86	N.D.	N.D.	N.D.	0.86		0.76
378	386	KLLEGEEER	1.10	1.11	1.26	0.98	1.11		0.98
379	386	LLEGEEER	N.D.	1.08	1.17	1.18	1.15		1.01
387	397	LRL$_{S}$P$_{S}$PTSQR	2.51	2.81	1.99	2.97	2.57		2.27
387	397	LRL$_{S}$PSPTSQR	0.39	0.68	N.D.	N.D.	0.53		0.47
389	397	LSPSPTSQR	0.95	0.62	N.D.	N.D.	0.79		0.70

402	417	ASSHSSQTQGGGSVTK	N.D.	N.D.	1.39	N.D.	1.39	1.23
420	427	KLESTESR	N.D.	N.D.	1.36	1.04	1.20	1.06
428	435	SSFSQHAR	N.D.	N.D.	0.95	N.D.	0.95	0.84
440	450	VAVEEVDEEGK	1.10	0.71	0.97	1.49	1.07	0.94
440	453	VAVEEVDEEGKFVR	1.02	1.22	1.03	1.26	1.13	1.00
458	470	SNEDQS$_{M}$GNWQIK	0.93	1.19	1.06	1.14	1.08	0.96
458	470	SNEDQSMGNWQIK	1.03	1.26	1.08	1.14	1.13	1.00
458	471	SNEDQS$_{M}$GNWQIKR	0.93	N.D.	N.D.	N.D.	0.93	0.83
471	482	RQNGDDPLLTYR	0.99	1.48	N.D.	N.D.	1.23	1.09
472	482	QNGDDPLLTYR	1.08	2.16	0.95	1.10	1.32	1.17
491	515	AGQVVTIWAAGAGATHSPPTDLVWK	1.12	N.D.	N.D.	N.D.	1.12	0.99
516	527	AQNTWGCGNSLR	1.05	1.16	1.02	1.16	1.10	0.97
528	541	TALINSTGEEVA$_{M}$R	1.14	1.19	1.07	1.11	1.13	1.00
528	541	TALINSTGEEVAMR	1.08	1.93	1.02	1.11	1.29	1.14
528	542	TALINSTGEEVA$_{M}$RK	N.D.	1.18	N.D.	N.D.	1.18	1.05

"S, T" stands for phosphorylation site and "M" stands for oxidation site.

N.D., Not detected
